# Impact of a shared decision-making mHealth tool on caregivers’ team situational awareness, communication effectiveness, and performance during pediatric cardiopulmonary resuscitation: study protocol of a cluster randomized controlled trial

**DOI:** 10.1186/s13063-021-05170-3

**Published:** 2021-04-13

**Authors:** Frédéric Ehrler, Cyril Sahyoun, Sergio Manzano, Oliver Sanchez, Alain Gervaix, Christian Lovis, Delphine S. Courvoisier, Laurence Lacroix, Johan N. Siebert

**Affiliations:** 1 Department of Diagnostic, Geneva University Hospitals, Geneva, Switzerland; 2 Department of Pediatric Emergency Medicine, Children’s Hospital, Geneva University Hospitals, 47 Avenue de la Roseraie, 1211 Geneva 14, Switzerland; 3 Division of Pediatric Surgery, University Center of Pediatric Surgery of Western Switzerland, Geneva University Hospitals, Geneva, Switzerland; 4 Department of Radiology and Medical Informatics, Division of Medical Information Sciences, Geneva University Hospitals, Geneva, Switzerland; 5 Quality of Care Unit, Geneva University Hospitals, Geneva, Switzerland; 6 University of Geneva, Geneva, Switzerland

**Keywords:** Situation awareness, Teamwork, Taskwork, Leadership, Mobile device, Information technology, Cardiopulmonary resuscitation, Pediatrics

## Abstract

**Background:**

Effective team communication, coordination, and situational awareness (SA) by team members are critical components to deliver optimal cardiopulmonary resuscitation (CPR). Complexity of care during CPR, involvement of numerous providers, miscommunication, and other exogenous factors can all contribute to negatively influencing patient care, thus jeopardizing survival. We aim to investigate whether an mHealth supportive tool (the Interconnected and Focused Mobile Apps on patient Care Environment [InterFACE]) developed as a collaborative platform to support CPR providers in real-time and share patient-centered information would increase SA during pediatric CPR.

**Methods:**

We will conduct a prospective, cluster randomized controlled trial by groups of 6 participants in a tertiary pediatric emergency department (33,000 consultations/year) with pediatric physicians and nurses. We will compare the impact of the InterFACE tool with conventional communication methods on SA and effective team communication during a standardized pediatric in-hospital cardiac arrest and a polytrauma high-fidelity simulations. Forty-eight participants will be randomized (1:1) to consecutively perform two 20-min video-recorded scenarios using either the mHealth tool or conventional methods. The primary endpoint is the SA score, measured with the Situation Awareness Global Assessment Technique (SAGAT) instrument. Enrollment will start in late 2020 and data analysis in early 2021. We anticipate that the intervention will be completed by early 2021 and study results will be submitted in mid 2021 for publication.

**Discussion:**

This clinical trial will assess the impact of a collaborative mHealth tool on increasing situational awareness and effective team communication during in-hospital pediatric resuscitation. As research in this area is scarce, the results generated by this study may become of paramount importance in improving the care of children receiving in-hospital CPR, in the era of increasing communication technology.

**Trial registration:**

ClinicalTrials.gov NCT04464603. Registered on 9 July 2020.

**Supplementary Information:**

The online version contains supplementary material available at 10.1186/s13063-021-05170-3.

## Background

Despite advances in resuscitation science and improvement of cardiac arrest (CA) survival over the past decades, only approximately 38% of children survive to hospital discharge after pediatric in-hospital cardiac arrest (p-IHCA). This number decreases to 6% to 20% after out-of-hospital cardiac arrest (p-OHCA) due to a non-traumatic cause [1, 2]. Survival after traumatic p-OHCA is even worse, with only 0.3% of children surviving to discharge with an intact neurological status [3]. Optimal technical skills influencing CPR quality are emphasized in resuscitation guidelines [4–8]. The American Heart Association (AHA) also highlights the importance of a team-based structure to optimize the management of critically ill patients to increase their chances of survival after cardiac arrest [9, 10]. However, the use of a team-based structure in critical situations does not ensure team performance and does not improve patient outcomes [11]. While rescuers have to assume individually assigned tasks, they should share a common and congruent goal-directed mental model. This implies effective non-technical cognitive and social skills to deliver optimal care, such as task management and coordination, team dynamics, leadership, SA, communication, and decision-making [9, 12–18]. Among these skills, SA and communication play a pivotal role.

SA refers to the individual’s ability to quickly gather pertinent information from the environment, unequivocal comprehension of its meaning, and the ability to project future events based on this comprehension [19]. This makes SA the main precursor to decision-making [20]. This concept can be extended to all team members, whereby each team member possesses complementary or shared SA required for their respective roles [21]. Poor SA during CPR can lead team members to be unaware of relevant information regarding the patient’s health clinical status, its implications, and the anticipation of patient requirements. This can impede the coordinated execution of CPR, impact on quality of care, increase the likelihood of errors and affect patient outcome [22, 23].

Effective, closed-loop, directed communication and information-sharing between team members is also essential during CPR. However, several disruptive factors can hinder their implementation, such as a noisy environment, interruptions, exogenous distractors, and the number of providers involved in various tasks [12]. A previous study demonstrated that ineffective communication among team members in critical care situations occurs in one-half of all events due to inaudible information or misunderstanding [15]. Moreover, non-team-leader-initiated communication (i.e., “outer-loop” communication) was observed in almost one-half of exchanges during CPRs, being potentially distracting for the team-leader and representing a lost source of information [24]. Hence, communication breakdowns and degradation of information-sharing are often attributable causes for adverse events and medical errors and also associated with degradation in SA [25]. This can directly impact morbidity and mortality [17, 26].

Current AHA resuscitation guidelines do not yet include specific recommendations to optimize non-technical skills during CPR. Similarly, in the Advanced Trauma Life Support (ATLS) course, there is little guidance on effective leadership, teamwork, or effective communication as a trauma team leader [27]. This partly relies on logistic barriers that make controlled and reproducible measurement of team interaction in real life-threatening trauma and cardiac arrest challenging [22, 28]. To overcome these limitations, in situ simulation-based studies offer the opportunity to assess non-technical skills in a standardized and controlled critical environment. Although previous organizational and training measures have been identified as possible means to improve team communication and SA during CPR [28–32], evidence is scarce regarding the impact of mobile health (mHealth) tools to support cardiac arrest team members. We developed a shared decision-making mHealth supportive tool (Interconnected and Focused mobile Applications on patients’ Care Environment [InterFACE]) as a collaborative platform to support CPR providers in real-time with shared patient-centered information.

### Previous work justifying this trial

**Fig. 1:**
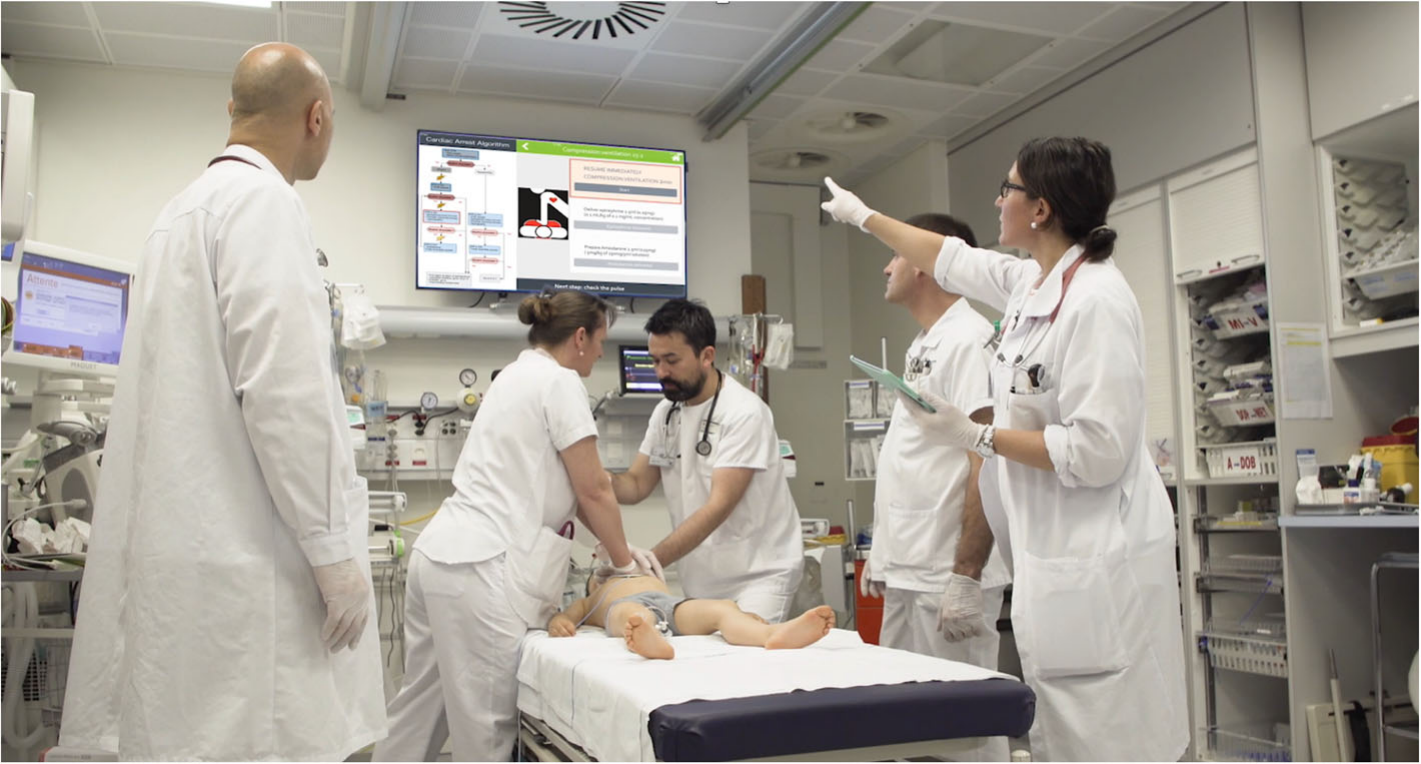
The InterFACE mHealth tool to be used during the forthcoming trial. The “Guiding Pad” mobile app installed on a tablet is communicating in real-time patient-centered resuscitation steps on the remote TV screen in the shock room. Informed consent for publication was obtained from the individuals (or their parent/legal) in Fig. 1

InterFACE consists of a dual, interconnected mHealth tool composed primarily of a mobile device app, namely “Guiding Pad”, interfaced to a remote large liquid crystal display (LCD) screen installed in the resuscitation bay and situated above the patient’s head (Fig. 1). In a previous multicenter, randomized crossover trial [33], we showed that medication errors, time-to-drug preparation, and time-to-drug delivery for continuous infusions during simulation-based, pediatric, in-hospital post-cardiac arrest scenarios were significantly reduced using the drug-dosing app PedAMINES, embedded into InterFACE. In another recent randomized trial [34], use of the “Guiding Pad” mobile app was associated with a shorter time to first and subsequent defibrillation attempts, reduced intravenous medication and defibrillation dose errors, and improved adherence to AHA recommendations compared with the use of pediatric advanced life support (PALS) pocket cards. However, it remains to be determined whether the implementation of the entire mHealth tool during pediatric CPR would improve SA, leadership, team communication, and performance. In this cluster randomized controlled trial, we will explore this assumption in a simulated model.

## Methods/design

### Objectives

The primary aim of this study is to investigate whether the mHealth tool improves SA among team members during standardized, high-fidelity p-IHCA and life-threatening polytrauma simulations. Secondary outcomes are the mHealth tool effect on leadership, team communication effectiveness, and performance compared with conventional communication methods. Our hypothesis is that the use of the tool might improve the above-mentioned non-technical skills, as well as clinical skills, when used by in-hospital emergency teams.

### Trial design and setting

This is a prospective, single-center, cluster randomized controlled trial that will be conducted in a tertiary pediatric emergency department (approximately 33,000 consultations/year) with voluntary pediatric emergency fellows, residents, and nurses. We will compare SA, leadership, communication skills, and team performance using the shared mHealth supportive tool (“InterFACE”, group A) or conventional team interactions (group B) during standardized p-IHCA and life-threatening polytrauma simulations. Participants allocated to group A will not be allowed to use any other cognitive support. Participants allocated to group B will be allowed to use the PALS pocket reference cards and a conventional calculator, but not any other cognitive support or mobile device app. No changes will be made to the app during the study.

**Fig. 2:**
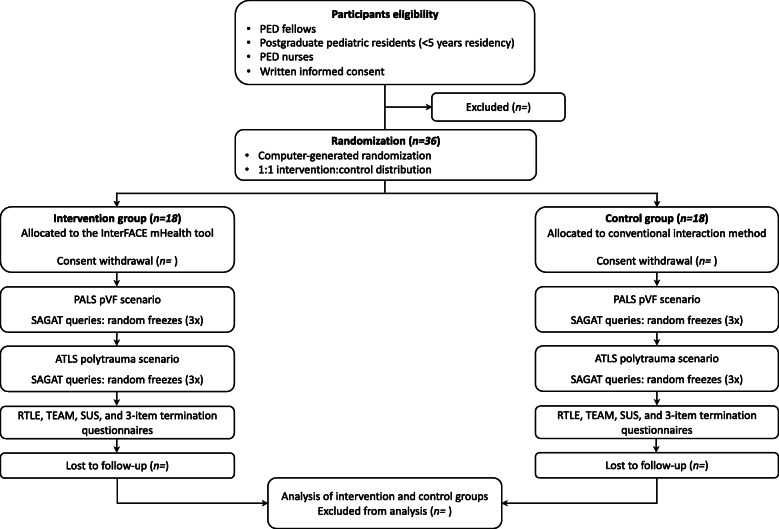
Trial flow chart

The development of the study protocol followed the Standard Protocol Items: Recommendations for Interventional Trials (SPIRIT) 2013 Checklist (Additional file 1) [35]. Figure 2 shows the trial flow chart and Table 1 the trial schedule. This trial protocol version 1.0 received a declaration of no objection by the Geneva Cantonal Ethics Committee on March 18, 2020, as the purpose of the study will be to examine the effect of the intervention on healthcare providers. The trial will be conducted according to the principles of the Declaration of Helsinki [36] and Good Clinical Practice guidelines [37].

**Table 1:** Standard Protocol Items: Recommendations for Interventional Trials checklist (SPIRIT) Figure. (T) SAGAT, (team) situational awareness global assessment technique; RTLE, resuscitation team leader evaluation; TEAM, team emergency assessment measure; SUS, system usability scale

	Study period				
	Pre-enrolment	Enrollment/consent	Pre-study baseline/allocation	2 × 20-min scenario	Close-out
Timepoint	***–t***_***2***_	***–t***_***1***_	0	t_**1**_	t_**x**_
**Enrollment**					
**Invitation to participate**	X				
**Eligibility screen**		X			
**Informed consent form**		X			
**InterFACE 15-min learning session**			X		
**Allocation**			X		
**Intervention**					
**Group A (mHealth tool)**				X	
**Group B (conventional method)**				X	
**Assessments**					
**Demographic data**			X		
**(T)SAGAT**				X	
**RTLE (leadership)**					X
**TEAM (teamwork, communication)**					X
**Time to 2nd outcomes**				X	
**Medication/defibrillation errors**				X	
**3-item termination questionnaire (10-point Likert scale)**					X
**SUS questionnaire**					X

### Participants and eligibility criteria

Any physician performing a pediatric emergency medicine fellowship in the pediatric emergency department (PED), as well as any postgraduate residents pursuing a < 5 years residency in pediatrics at Geneva University Hospitals (Geneva, Switzerland), will be eligible. Registered nurses with a specialty in pediatric emergency nursing will be eligible. Inclusion criteria are having followed a standardized 15-min introductory course on the use of the InterFACE tool and being willing to grant written informed consent. Participation to a simulation in the past month is an exclusion criterion to avoid a recent training effect (knowing that in our PED, nurses, and physicians participate in about one simulation every 3 months).

### Informed consent process

On the day of participation, in the presence of the study investigators, participants will provide written informed consent. Important concepts will be highlighted via bulleted text. A checkbox will assess whether participants understand key consent information. These consent forms will be collected and countersigned by the study investigators and stored securely in a locked room at the University Hospitals of Geneva. If the study investigators have reason to believe that a participant is not aware of study requirements or may be unable to provide informed consent, they will notify the participant that they will not be enrolled.

### Recruitment process

To achieve adequate subject enrolment to reach the target sample size on the day of participation, teams will be randomly recruited and scheduled weeks before the start of the study by a blinded non-investigator. To avoid preparation bias, they will be informed of the upcoming simulation study, but not of its purpose and outcomes. In the case of participant unavailability, the research team will organize a replacement. As basic life support training is a requirement for caregivers at our institution, all participants will have previously completed this course prior to study entry. All nurses will be assumed to have an equivalent competence with intravenous drug preparation and dose calculation as this is part of their regular practice and training background. Study participants will not be involved in the study design, choice of outcome measures, or the execution of the study. No participant will be asked for advice on the interpretation or writing of the study results. All participants will be informed of the results after study completion.

### The InterFACE tool

InterFACE was developed at the Geneva University Hospitals following an iterative user-centered process and ergonomic-driven approach by senior pediatric emergency physicians and nurses, computer scientists, software developers, and ergonomists with a strong background in medical app development. On the basis of pediatric resuscitation observations, AHA and ATLS guidelines, and focus groups, the team worked closely together to identify the key functionalities and processes to be implemented [38]. Both main components—the “Guiding Pad” app and the remote screen—are part of the whole shared decision-making tool intended to support and guide CPR providers during resuscitation, while improving team SA.

**Fig. 3:**
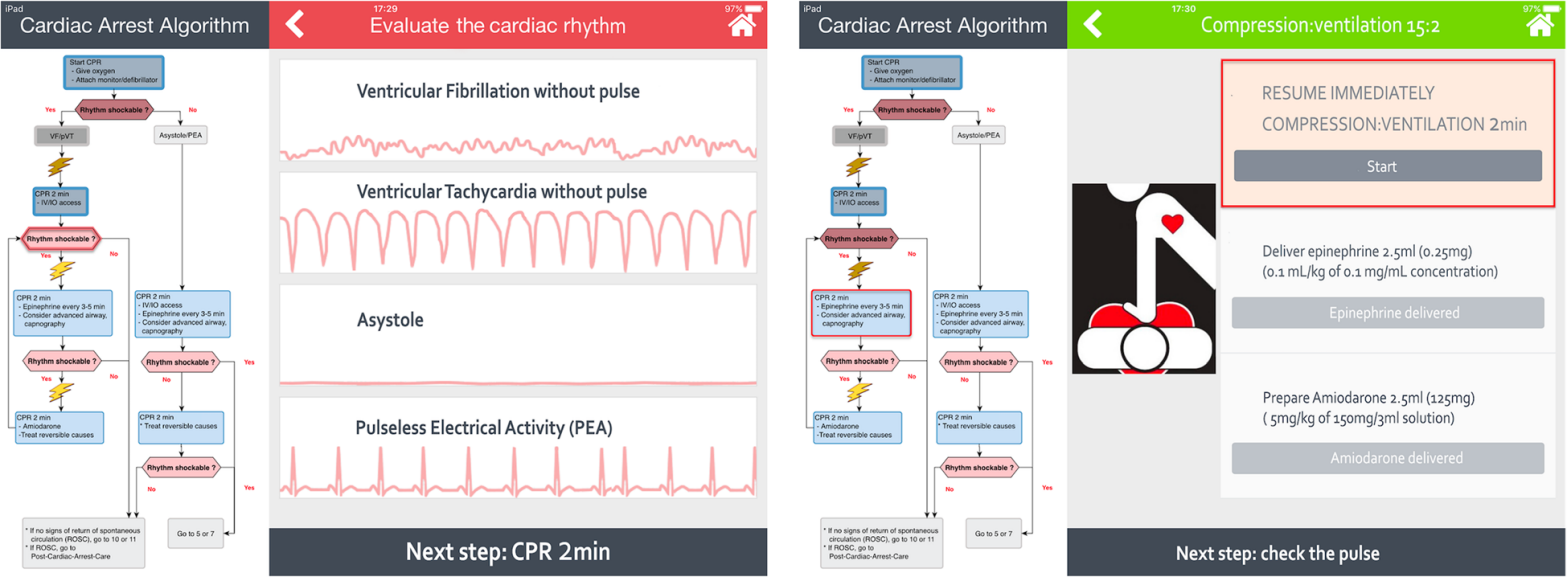
“Guiding Pad” screenshots. Two screenshot examples of the Guiding Pad app. The left-hand side of the screen displays the AHA pediatric advanced life support (PALS) ventricular fibrillation (VF) and pulseless ventricular tachycardia (pVT) cardiac arrest algorithm. The current step (e.g., determining the shockable status of the arrhythmia) of the resuscitation process is surrounded by a blinking red line. Past actions already accomplished are shown as shaded. At the top right-hand side of the screen, a color-coded title depicts the current step in progress. On the right-hand side of the screen, the sequence of actions to be taken (e.g., to select the right pulseless dysrhythmias under consideration [left screenshot]) are displayed in a stepwise manner to facilitate accurate progression along the algorithm. The current action (e.g., to resume compression and ventilation [right screenshot]) is brought to the attention of the provider by a red-box warning and requires validation by a simple click. Once completed, the next action will be to deliver the weight-based epinephrine dose automatically calculated by the app and then to prepare amiodarone). At the bottom right-hand side, a footer helps to anticipate the next cardiopulmonary resuscitation step. CPR: cardiopulmonary resuscitation; IO: intraosseous; IV: intravenous; PEA: pulseless electrical activity

The “Guiding Pad” app was developed using Angular version 8, a development framework created by Google to build mobile and web apps. Assessment of this app and details regarding its functionalities have been described in a previous randomized trial [34]. In brief, the app displays a split screen with PALS algorithms split into interactive stages and adapted for tablet devices, as well as patient-centered cognitive aids to help with real-time decision-making (Fig. 3). The user can easily navigate through the algorithm in use and across the multiple algorithms at any time. For the purpose of the trial, no specific trauma guidelines will be implemented within the app. Indeed, the ATLS guidelines follow substantially the same didactic approach as outlined in the AHA guidelines for patients with primary cardiac arrest, with specific attention given to the technical support of vital functions with trauma-related equipment. Therefore, the app allows to select an exhaustive pre-defined and organized list of resuscitation materials and trauma-related equipment (and also symptoms and conditions that can be encountered during CPR). Finally, weight-based drug doses are automatically calculated by an on-board engine inherited from another evidence-based app [33].

**Fig. 4:**
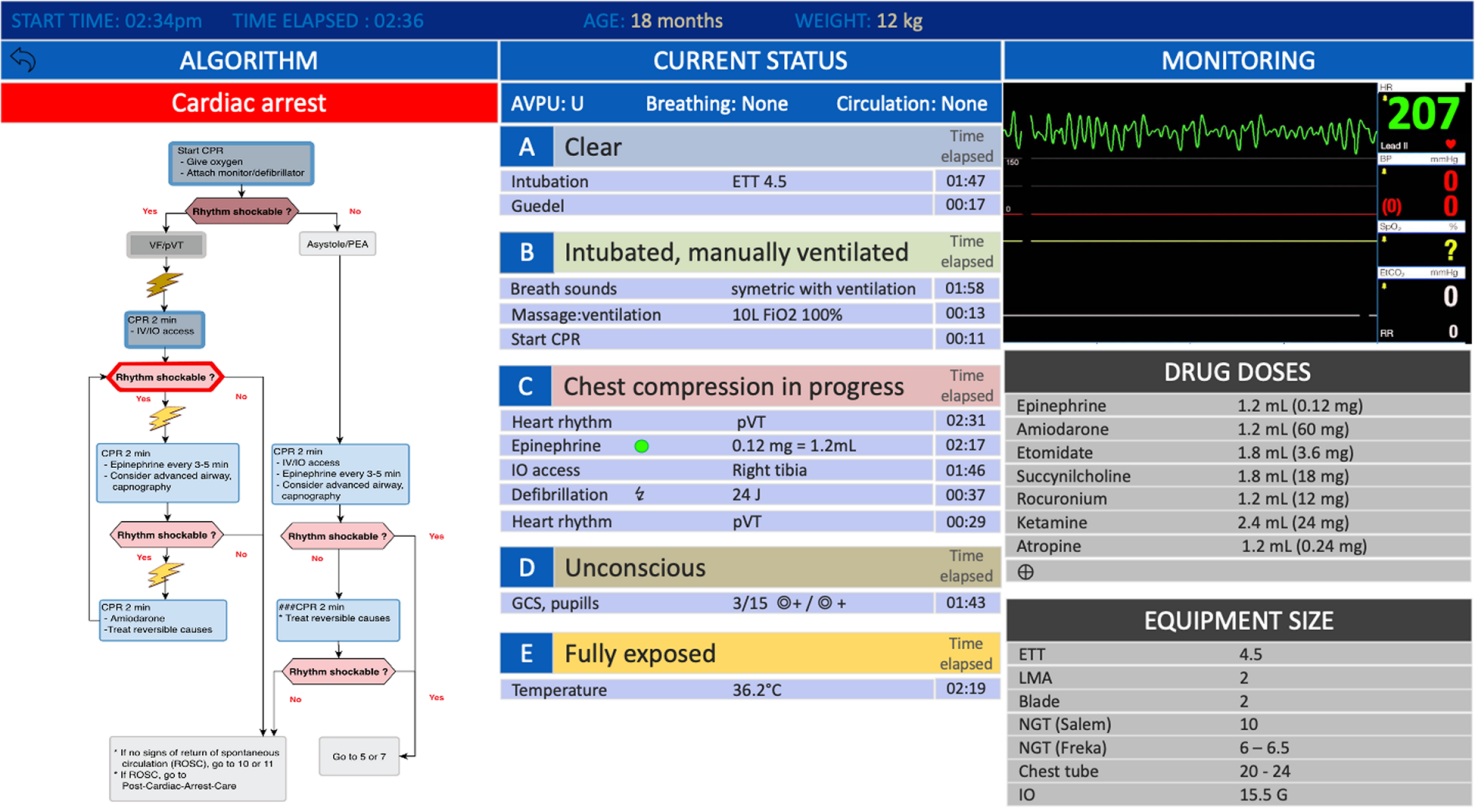
InterFACE screenshot of the integrated LCD screen display. The information is displayed in windows that can be flexibly arranged in different layout positions and sizes around the screen, according to user preference. Each window contains patient-centered real-time information, displayed in chronological order and following the systematic ABCDE approach, including vital signs, identified pathology, interventions (procedures, medication received, etc.), as well as resuscitation algorithms. The first section on the left-hand side of the screen displays an entire algorithm overview, thus allowing users to situate the stepwise resuscitation progress in real time along that algorithm. The current step of the resuscitation process is surrounded by a blinking red line that allows an immediate understanding of the current position within the algorithm. Each action already performed turns gray. However, the tool provides the possibility for users to navigate back and forth through the algorithm (or other algorithms) at any time in order to select one of the resuscitation steps if needed. The right-hand side of the screen displays a detailed and clickable list of drug doses and equipment size at user’s disposal and intended as cognitive aids helping with decision making

All critical actions performed by the team are automatically validated on the mobile app and reported on a remote LCD screen above the patient’s head in real-time via a dedicated and secured internal WiFi connection (Fig. 1). The pertinent information gathered through the mobile app, as well as the patient monitor, is therefore displayed in the sight of the entire resuscitation team to be shared in an easy-to-read and spatially organized manner, sorted in chronological order, and paralleling the CPR in progress (Fig. 4). According to the concept of the proximity compatibility principle to display design [39], information relevant to a particular resuscitation task is rendered close together within a dedicated window. All actions undertaken by the providers are saved in log files and can be retrieved at any time for debriefing or medical chart documentation purposes. Finally, the app’s architecture is flexible enough to accommodate additional information sources as future needs arise.

### Intervention and resuscitation scenarios

Each team will comprise six participants: (1) a team leader (fellow in pediatric emergency medicine), (2) a physician (resident in pediatrics) undertaking the primary/secondary surveys and performing the procedures, (3) a dedicated trained assistant for airway management, (4) a dedicated trained assistant for chest compressions, (5) a nurse for vascular access and drug preparation/administration, and (6) a scribe (second nurse) to document events and actions in a chronological fashion (either with the app or conventional methods). As PALS instructors and senior physicians, the study investigators will not interfere in the conduct of the CPR (except in the case of a significant deviation from the algorithm that must be followed to achieve ROSC; which will then be regarded as an error). Among them, a study investigator will play the role of on-call hospital specialists and radiologists, if needed. A second study investigator will operate the simulator and a third will freeze the situation at certain defined timepoints and provide the scripted questions.

**Fig. 5:**
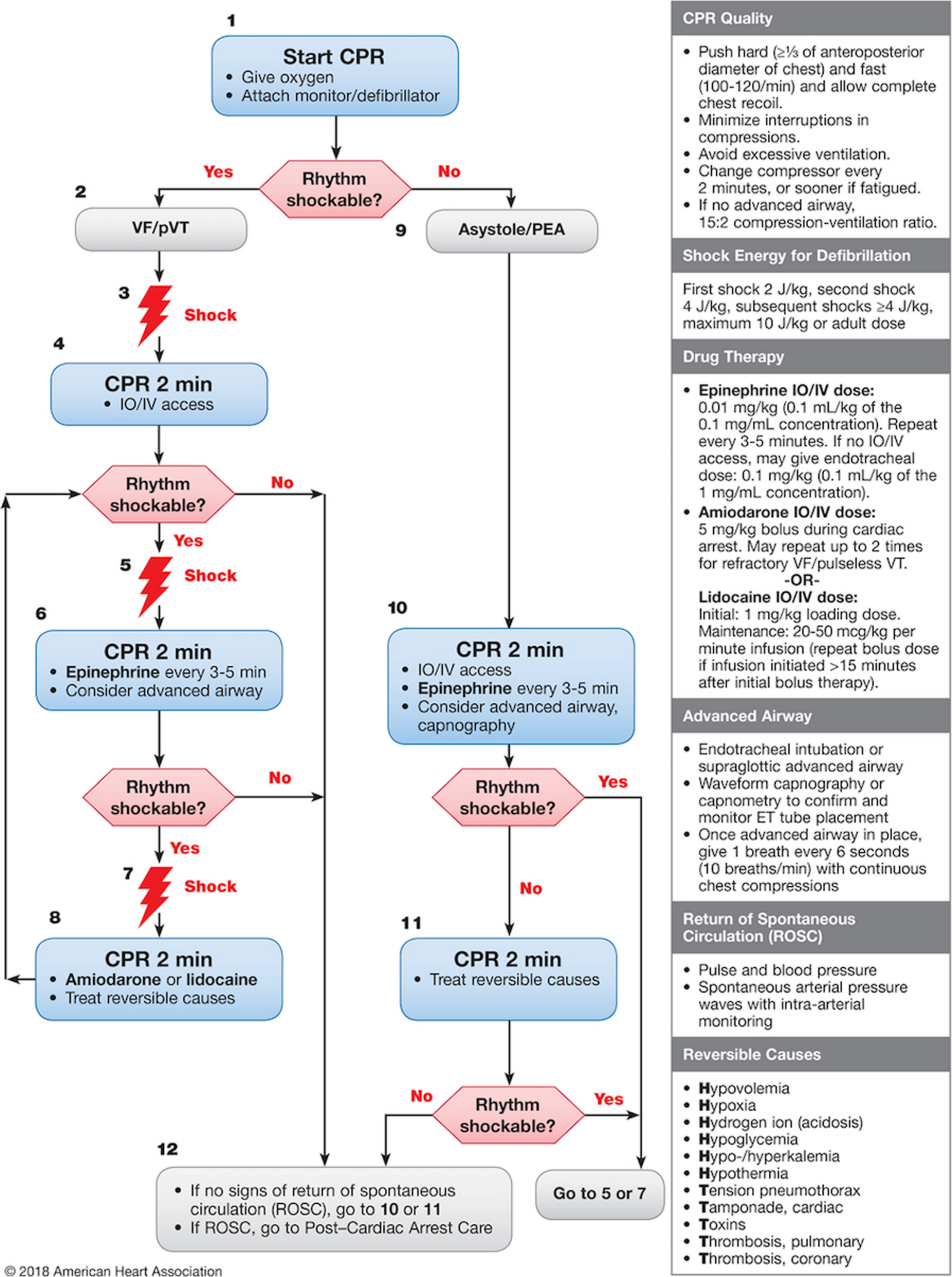
AHA pediatric cardiac arrest algorithm – 2018 update [5]

On the day of participation, each subject will complete an anonymous survey about basic demographic information, professional length of medical training, PALS and ATLS training, and simulation and computer experience. After random allocation, each team will receive: (1) a standardized 15-min training session on how to use the app, (2) a presentation of the simulation manikin’s characteristics, and (3) the topics of three assessment questionnaires (see below), but to prevent unblinding, not the queries themselves. The teams will then be asked to consecutively perform two 20-min highly realistic, scripted CPR scenarios on a high-fidelity WiFi manikin (Laerdal SimBaby, Laerdal Medical, Stavanger, Norway). High levels of realism are known to immerse participants in the simulated experience and prevent confounding variables that might potentially affect the way they perform [30]. Within each scenario, three separate “freeze” periods will occur at random points in time to assess the shared and complementary SA of each team member individually regarding the CPR in progress. During freezes, the entire team will have to turn away from the patient, monitors and app to “blank out” the simulator and avoid any cognitive help from the simulated environment that could otherwise bias the respondent’s answers to the questionnaires. The scenarios will be standardized to strictly follow the 2018 AHA pediatric ventricular fibrillation algorithms [5] (Fig. 5) and the 2018 ATLS guidelines [40]. They will be extensively tested before the trial to achieve optimum functioning on the day of participation and performed on the same high-fidelity manikin already primed with vital signs appropriate for the scenarios. Both scenarios will be completed in the same order and the procedure will be standardized across all teams to follow the same chronological progression and range of difficulty in order to ensure that each participant is exposed to exactly the same case, with similar challenges in technical and non-technical skills. The uniform delivery of the scenarios throughout the entire study will minimize confounders. Study investigators will allow progression through the scenarios only once predefined milestones have been reached, irrespective of error occurrence or the time taken to achieve them.

The scenarios will be conducted in situ in the PED resuscitation bay to increase realism, thus allowing participants to make use of real resources in the actual environment where they are expected to perform CPR. The decision to use or not use any equipment will remain personal, as in real-life situations. All participants in group B will be offered the possibility to hold PALS pocket reference cards in their hands throughout the entire scenarios. Whether they refer to these or not will be left to their entire discretion, similar to real-life settings. The locale will be exclusively devoted to the simulation to prevent unexpected interruptions or external stimuli. Monitoring alarms will be activated to increase realism and stress, and distracting exogenous factors (telephone call, immediate unavailability of specialists, non-essential information, etc.) will occur.

The untimed portion of the simulation will start by turning on the two video cameras (GoPro Hero 7 Black edition; San Matteo, CA, USA) with the team awaiting 10 m away from the resuscitation bay. Participants will then be invited to enter the bay by a study investigator. When entering the bay, a clinical statement to recognize the life-threatening condition of the patient, including his exact weight and age, will be given by the investigator as follows: “Here is Cindy, a 12-kg, 18-month-old girl known for a congenital type 2 long QT syndrome who experienced a witnessed syncope after she suddenly woke up from a nap in her stroller after a loud car horn. She was brought to the emergency department by her parents without prior resuscitation maneuvers and immediately installed in the resuscitation bay as she is unconscious, pale and not breathing”. At this moment, the investigator will ask the team leader to manage the situation with the team, either with the support of InterFACE or conventional methods. According to PALS recommendations, the team will have to start the CPR by assessing the patient with the pediatric assessment triangle [41]. Because of the invariable absence of a pulse in an unconscious and non-breathing child, the team should follow the circulation-airway-breathing approach and start a 2-min cycle of chest compressions and ventilations (15:2 ratio) and use the resuscitation equipment. Goals for this scenario will be to recognize a ventricular fibrillation rhythm and to adhere to the PALS cardiac arrest algorithm. ROSC will ensue after a bolus of 5 mg/kg amiodarone and the subsequent fourth electric shock. After ROSC recognition by the team, the scenario will end and the GoPro cameras turned off.

The team will be asked to exit the bay to have a 10-min rest period in a quiet room, free of cognitive information/tasks. The leader will have to wait separately in another room.

The whole team will then be asked to enter the resuscitation bay for the second scenario adapted from Crozier et al. [42]. When entering the room, the clinical statement will be: “Here is Junior, a 13-kg, 20-month-old boy involved in a high velocity motor vehicle accident 30 min ago. He was brought to the ED with an altered state of consciousness and pallor. A cervical spine collar is in place and the intravenous line was dislodged when he was transferred from the stretcher to the bed”. At this moment, the investigator will ask the team leader and team members to manage the situation similarly to the previous scenario, using InterFACE or conventional methods. They will be expected to progress through the primary and secondary surveys according to ATLS guidelines. Goals for this scenario will be to recognize a hypotensive hypovolemic shock secondary to major blood loss from internal and external injuries and to recognize the signs and symptoms of a closed head injury. The scenario will end and GoPro cameras will be turned off after the patient’s return to a normotensive state following saline boluses and blood transfusions and the decision to perform a computed tomography scan. Moulage (bruises, external blood hemorrhage) will be applied to the manikin in the second scenario to enhance physical findings. Neither pilot testing nor repetitions will be permitted. There will be no educational adjuncts prior to or after the study period. At the study close-out, participants will be instructed not to disclose any information from the scenarios to other participants to prevent any preparation bias.

### Outcome measures

Research using simulation as a valid and reliable investigative methodology to study factors affecting human and systems performance in health care has been reviewed [43]. In this simulation based-study, outcomes will be evaluated by several instruments. Raters will be trained to use the rating instruments in their native language prior to their use. A schematic diagram of the time schedule of data collection for all outcome measures is shown in Table 1. To report the primary and secondary outcomes, we will follow the five levels of specification in reporting outcome measures defined in [44, 45] that include information about the (1) the domain, (2) specific measurement, (3) specific metric to characterize each participant’s results, (4) the method of aggregation, and (5) the time frame that will be used for analysis.

### Primary outcome

#### Situational awareness

Direct measures of SA through the SA global assessment technique (SAGAT) instrument were found to be a highly sensitive, reliable, and predictive measure of individual and team performance in a patient-simulated environment [21]. In this trial, SAGAT questionnaires representing the three SA levels (i.e., perception, comprehension, and projection) were adapted from Coolen et al. [19] by an expert panel of senior pediatric emergency physicians, nurses, and a statistician using a goal-directed task analysis (GDTA) methodology as described by Endsley et al. [46] (Additional file 2). SA (domain) expressed as a score in absolute value in conjunction with percentage (metric) will be measured using the SAGAT (specific measurement) during video review of each scenario. Both the individual participant SAGAT score and the team SAGAT score, defined as the degree to which each team member possesses the SA required for their role and calculated as the sum of individual SAGAT scores, will be calculated [42, 47]. The SAGAT scores will be further divided into their three dimensions to individually evaluate perception, comprehension, and projection of each team member and to compare them to what truly happened during the simulation. This will be done similarly for the TSAGAT. According to Endlsey et al. [46], the SAGAT responses for categorical variables will be scored as either correct (1) or incorrect (0) by two of the investigators. For numerical responses, answers will be considered correct based upon a 10% pre-defined tolerance range settled by the research investigators around the true value. Higher SA scores will denote higher SA. Mean overall scores will be aggregated to compare both study arms (method of aggregation). The time frame of interest for this endpoint is over the duration of the whole CPR scenario.

Practically, each scenario will be frozen three times (2-min duration per freeze; queries’ length will be compatible with time constraints) at random points in time during the scenario to issue queries chosen randomly from a predefined list (Additional file 3). Random freezes have been shown not to impact on participant performance measures [46] and to limit recall bias that could otherwise arise if queries are asked at the end of the simulation [21]. This will allow to assess respondents’ awareness of the current situation so that query occurrence will not be associated with the occurrence of specific related events [46]. During each freeze, participants will be asked to complete a total of eight multiple choice queries for level 1, four for level 2, and four for level 3 of SA in a written confidential manner. We will therefore collect per scenario a total of 768 SAGAT queries and eight TSAGAT, with a range of possible scores of 0 to 16 on each SAGAT questionnaire and a combined total score of 0 to 96 on TSAGAT. Over the course of the experiment, this will represent a total of 96 SAGAT (768 queries) and 16 TSAGAT. No freezes will occur earlier than 5 min after the beginning of the scenario to allow participants to build up a representation of the situation [46]. The second and third freezes will be interspaced at least 1 min from each other and from the first freeze.

### Secondary outcomes

#### Leadership

Leadership (domain) will be measured with the resuscitation team leader evaluation (RTLE) instrument (specific measurement) [48]. This instrument developed by Grant et al. encompasses four leadership concepts: (1) the physical and verbal leader’s position, (2) communication and delegating skills, (3) ability to assess, adapt and anticipate, and (4) ability to ask for internal and external help [48]. It comprises 12 items rated on a 4-point Likert scale (metric) from 0 (not performed) to 3 (performed well, consistently) to score leadership and communication skills (Additional file 4). A not applicable (N/A) category is also available for those items not relevant to the scenario. For the purpose of this trial, we will remove items 1 and 12 as the leader will be clearly identified from the beginning of the scenario and teams will not be advised to ask for external help. Therefore, the total item score expressed as an absolute value will range from 0 to 30. Mean overall scores will be aggregated to compare both study arms (method of aggregation). The time frame of interest for this endpoint is over the duration of the whole CPR scenario.

#### Team performance

Leadership, communication skills, team work, task management, and the team’s overall performance (domains) will be measured as scores obtained from the team emergency assessment measure (TEAM) instrument (specific measurement) [49]. Although a number of tools are available for assessing teamwork performance, the TEAM stands out as the most valid and reliable instrument to use in emergency departments [50]. TEAM comprises 11 items rated on a 5-point Likert scale from 0 (never/hardly) to 4 (always/nearly always), which are summed up into a total item score (metric) expressed as an absolute value ranging from 0 to 44 [49]. The final score obtained allows to assess the performance of the emergency medical team based on three categories: leadership (items 1 and 2); teamwork (items 3 to 9); and task management (items 10 and 11). Items 8 and 9 relate to SA perception and projection, respectively. Furthermore, the team’s overall performance is rated through a twelfth item on a global rating scale of 1 to 10 (higher score denotes better performance) (Additional file 5). Mean overall scores will be aggregated to compare both study arms (method of aggregation). The time frame of interest for this endpoint is over the duration of the whole CPR scenario.

#### Time to critical life-saving maneuvers

Team effectiveness (domain) will also be measured on video reviewing (specific measurement) as the time spent in seconds by the team to achieve the pre-designed goals set out by the AHA for the PALS course and the American College of Surgeons for the ATLS course [10, 51, 52]. They will comprise the elapsed time in seconds between the end of the clinical statement by the study investigator to (1) ventricular tachycardia or hypotensive shock recognition; (2) initiation of chest compression; (3) time to each defibrillation attempt; (4) time to administration of intravenous drugs, volume expansion with 0.9% sodium chloride or blood transfusion; and (5) time interval between defibrillation attempts (metrics). If not achieved within the 40-min scenario, this will be considered as a lack of goal completion. It should be noted that for the trauma scenario, we decided not to use the modified non-technical skills scale for trauma (T-NOTECHS). This decision is based on the serious risk of assessment bias with this instrument, as emphasized by the consensus-based standards for the selection of health measurement instruments (COSMIN) [50]. Mean times per goals will be aggregated to compare both study arms (method of aggregation). The time frame of interest for this endpoint is over the duration of the whole CPR scenario.

#### Medication dosage errors

Errors in drug dose administration (domain) in milligrams will be measured on video reviewing (specific measurement) both as the percentage and absolute deviations from the 2018 AHA PALS cardiac arrest algorithm (metric). Errors will be also measured both as the percentage deviation from the amount of delivered drug compared with the correct weight dose as prescribed by the physician and the absolute deviations from that dose (metric). For the second scenario, errors in fluid resuscitation volumes (in milliliters) will be measured as a deviation from the 2018 ATLS guidelines (metric). An initial 20 mL/kg bolus of isotonic crystalloid followed by 10–20 mL/kg of packed red blood cells and 10–20 mL/kg of fresh frozen plasma and platelets are expected [40]. Moreover, defibrillations (in Joules) and the number of shocks will be also measured during the first scenario (metric) [50]. Mean error rates will be aggregated to compare both study arms (method of aggregation). The time frame of interest for this endpoint is over the duration of the whole CPR scenario.

#### System usability scale (SUS)

At the end of each evaluation, we will measure the participants’ perceived usability (domain) of InterFACE using the system usability scale (SUS) questionnaire (specific measurement) designed by Brooke [53]. It consists of a 10-item questionnaire with five response options for each item (metric), based on their level of agreement ranging from 1 (strongly disagree) to 5 (strongly agree). Following Brooke’s scoring system, for odd-numbered statements 1, 3, 5, 7, 9 (positively-worded items), the score contribution is equal to the scale position minus 1 (e.g., strongly agree: 5–1 = 4). For even-numbered statements 2, 4, 6, 8, 10 (negatively-worded items), the score contribution is equal to 5 minus the scale position (e.g., strongly agree: 5–5 = 0). Each score contribution will fall within the range of 0 to 4. The participant’s scores for each item are then added up together and multiplied by 2.5 to convert the original scores of 0–40 to 0–100. Although the scores are 0–100, these are not percentages of usability and should be considered only in terms of their percentile ranking. To obtain a SUS score of 100, the respondent must answer five to all odd questions and 0 to all even questions. It is generally considered that a score of 75 and above is good, while a score of 50 and 75 is fair. A score below 50 reveals strong disagreement in terms of satisfaction. The mean SUS score in the intervention group will be used to assess the overall perceived usability of InterFACE (method of aggregation). The time frame of interest for this endpoint is over the duration of the whole CPR scenario.

#### Perceived stress and satisfaction

A three-item questionnaire using a 10-point Likert scale will be administered to participants (specific measurement). It will measure: (1) the stress perceived before the scenario starts (“on a scale from 1 to 10, how stressed are you now?”); (2) the overall stress perceived at the end of the scenario (“on a scale of 1 to 10, how stressed (maximum reached) were you during the drug preparation period?”); and (3) the satisfaction about the tools used during the resuscitation scenario (“on a scale of 1 to 10, how satisfied were you with your preparation experience?”) (domains). The scales range from 0 (minimum score) to 10 (maximum score), increments are integers between 0 to 10 (metric). For stress, higher values represent a worse score, whereas for satisfaction higher values represent a better score. No subscales will be combined. Stress and satisfaction scores will be expressed as percentages in conjunction with absolute n/N values. Mean scores will be aggregated to compare both study arms (method of aggregation). The time frame of interest for this endpoint is over the duration of the whole CPR scenario.

### Data collection and management

Data collection will be carried out by the responsive simulator detectors (Laerdal SimBaby) and the two GoPro video cameras. The set-up of both cameras will be standardized to record at a resolution of 1080p at 60 frames per second, a wide field of view, and a 16:9 aspect ratio. Similarly, the position of the cameras will be standardized. The first camera will be placed over the manikin’s head, below the LCD screen, to allow to capture footage of the whole resuscitation scene. The second camera will be placed on a tripod, 2 m away from the manikin’s feet, where the leader will stand to film the scene from the leader’s point of view (including a view on the patient monitor). The recorded videos will be safely stored in duplicate on secured hard-disk drives in a locked room at the Geneva University Hospitals. As all scenarios will be fully video-recorded, all actions, communications, and interactions will be later scored by two raters to allow for the calculation of interrater reliability. The length of the freezes will be measured using the video recordings and deducted from the whole scenario duration to allow accurate measure of the different timed outcomes. All actions performed with the app will be automatically saved locally in log files for further analysis. This study offers the major advantage to observe a unique 60-min period per resuscitation team. Therefore, neither follow-up nor retention plans will be necessary. The resuscitation algorithm is highly standardized and deviation from the algorithm is a parameter of interest in our study. Data collection will be carried out using the REDCap database (REDCap, Vanderbilt University, Nashville, TN, USA; https://www.project-redcap.org/resources/citations/) by trained investigators.

### Data monitoring committee

A Data Monitoring Committee (independent from the trial funder) composed of FE, DC and JNS will verify the correctness of data collection, manage them, and secure their encoding in the REDCap database on secured servers hosted at the University Hospitals of Geneva.

### Steering committee

A Steering Committee composed of CS, SM, OS, DC, LL, and JNS will monitor the day-to-day trial conduction with weekly meetings scheduled throughout the duration of the project to keep up to date with progress and ensure coordination of the data monitoring committee. The committee will oversee and manage the study to ensure that participant enrolment, allocation groups assignment, and participation are conducted according to the trial protocol.

### Power and sample size calculation

A previous study found a 23.5% SA improvement in intensive care unit nurses exposed to an integrated information display compared to a traditional display exposure [54]. In this trial, the sample size was calculated to provide 90% power at a two-sided alpha level of 5% in detecting a relative difference of at least 25% SA improvement between intervention groups, with an assumed standard deviation (SD) set at 0.2. This SD is based on and conservatively about twice as much as that observed by Koch et al. [54]. The required sample size is 14 participants per study arm. To prevent a potential loss of power due to misspecification of assumptions, 18 participants (= 3 teams of 6 people per team) should be recruited per randomized group. To take into account the cluster randomized trial design, this sample size was inflated using a variation inflation factor of 1.25 (= 1 + 0.05(6–1); 0.05 being the intraclass correlation coefficient). The total sample size (i.e., 45 participants) was finally rounded up to 48 participants to obtain 8 teams of 6 participants per team.

### Group allocation

Participants will be randomized using a single, constant 1:1 allocation ratio determined with web-based software (http://www.sealedenvelope.com). Selection criteria will be checked prior to participation in the study.

### Blinding

Blinding to the outcomes will be maintained during recruitment to minimize preparation bias. Allocation concealment by the data analyst (DC) will be ensured with the allocation software and will not be released until the participants start the scenario. A post-scenario video review will be done without blinding by two reviewers, but undertaken independently with each reviewer blinded to the other’s reviews. In the case of disagreement, a third independent evaluator will help reach a consensus. The data analyst (DC) will not be blinded to the intervention.

### Confidentiality

Information about study subjects will be kept confidential. All data will be entered into the REDCap data management system where all data on study participants are assigned an individual identifying code that does not contain identifying information. Only anonymized data will be submitted as part of the statistical analysis. No direct or indirect personal participant’s study information will be released outside of the study without his/her written permission. Individual performance during the resuscitation scenario will remain confidential and will not be communicated at the institutional level.

### Statistical analyses

For the primary outcome, 48 subjects completing two scenarios each will provide a total of 96 SAGAT and 16 TSAGAT scores for analysis. For each query, two study investigators will score the answers. In the case of disagreement, a third investigator will find a consensus. The normality of variable distributions will be examined with the Shapiro-Wilks test. For normally distributed variables, means and SDs with 95% confidence intervals (CI) will be reported and scores compared between intervention groups using Generalized Estimating Equations (GEE) to account for the cluster randomized design. Non-normally distributed variables will be reported with medians and interquartile ranges.

For the secondary outcomes, each of the three SAGAT domains will be compared between the intervention groups using GEE for each scenario. We will also compare SAGAT scores according to the team members’ roles. Regarding the RTLE and TEAM instruments, differences between the two intervention groups will be assessed using multinomial logistic GEE for the single items, and linear GEE with an identity link for the sum score, and the global rating performance.

Kaplan–Meier curves for the time elapsed between the end of the clinical statement by the study investigator and timed secondary outcomes will be compared using the log-rank (Mantel-Cox) test including a cluster term to account for the cluster randomized design. Wrong defibrillation or drug doses will be measured as a deviation from the amount of energy delivered in Joules or drug doses in milliliters compared to 2018 AHA PALS cardiac arrest algorithm, respectively. Use of epinephrine 0.1 mL/kg (0.1 mg/ml concentration) and amiodarone 5 mg/kg (150 mg/3 mL concentration) are expected for the first scenario. We define an emergency medication dose administration error as a deviation from the correct weight dose of more than 10% [55]. Moreover, defibrillations (in Joules) and the number of shocks will be also measured during the first scenario. Defibrillation doses of 2 J per kg for the first attempt and 4 J per kg for the subsequent second, third and fourth attempts are expected [5]. A GEE model with a Poisson link will be used to assess the relationship between absolute errors in defibrillation and drug doses expressed as categorical variables. Wrong defibrillation mode will be also measured. Absolute deviations will be analyzed. The mean (SD) difference obtained with each intervention method will be reported with a 95% CI. Univariable linear GEE with 95% CI will be performed to assess whether time to initiation of chest compression, defibrillation attempts, and drug delivery are associated with prior resuscitation experience as a clinician in real-life and simulated environments. Similar analyses will be carried out for errors in fluid resuscitation volumes (in milliliters) and blood transfusion during the second scenario. An initial 20 mL/kg bolus of isotonic crystalloid followed by 10–20 mL/kg of packed red blood cells and 10–20 mL/kg of fresh frozen plasma and platelets are expected [40].

A first rater (one of the study investigators) will review all videos. To assess the reproducibility of the video review procedure, a second reviewer will independently duplicate the review in a random sample of 20% of all videos. Interrater reliability scores on video reviewing will be calculated using Cohen’s kappa coefficient for categorical variables. We define poor reliability as a kappa coefficient of < 0.4, fair reliability as 0.4–0.6, good reliability as > 0.6–0.8, and excellent as > 0.8. Any disagreements in video reviewing will be resolved by a third independent investigator, including appropriate revision of the implemented reviewing strategy, followed by double-reviewing of a further 10% until reliability on video reviewing (a kappa of 0.6 or greater) is achieved. For continuous variables, the Bland-Altman method will be used to plot the difference of values reported by both reviewers against the mean value for each outcome. The limits of agreement will be assessed by the interval of ± 1.96 SDs of the measurement difference on either side of the mean difference. The null hypothesis that there is no difference on average between both reviewers will be tested using a *t* test. The mean difference will be reported with its 95% CI. Additionally, the intraclass correlation coefficients for the volumes of drugs drawn, time to drug preparation, and time to drug delivery will be assessed, assuming that raters are a sample from a larger population of possible raters.

Finally, means and SDs will be determined for perceived stress and satisfaction scores of participants for each scenario, as well as for the SUS questionnaire, and reported with descriptive statistics. In the case of missing data, a complete case analysis will be conducted. No multiple imputations are planned. All statistical tests will be two-sided with a type I error risk of 5%. Data analysis will be performed using GraphPad Prism, version 7 (GraphPad Software, San Diego, CA, USA) for graph figures, and R version 4.0.4 (R Foundation, Vienna, Austria) for descriptive statistics and statistical tests.

### Dissemination policy

Final results of the trial will be reported in accordance with the Consolidated Standards of Reporting Trials of Electronic and Mobile Health Applications and Online TeleHealth (CONSORT-EHEALTH) [56] guidelines and the Reporting Guidelines for Health Care Simulation Research [57]. It is our intention to present these at scientific congresses and to publish the results in an international peer-reviewed journal, irrespective of the magnitude or direction of effect. The investigators of the research team will be named coauthors on all future related publications.

## Discussion

Pediatric CA is a high-risk low-frequency event associated with death or severe neurological sequelae in survivors. Recent studies show that p-IHCA affects 7100 to 8300 children per year in the USA [58], of which 14% occur in PEDs [59]. By contrast, p-OHCA accounts for a further 7037 children brought to United States PEDs each year [1]. Variable adherence to resuscitation algorithms is commonly observed and can hinder optimal trauma and CPR care, thus jeopardizing patient outcome and survival [60–64]. In addition to the technical skills of individual rescuers, shared mental non-technical skills at the team level such as SA, collective team interaction patterns, leadership, and dynamic decision-making affect adherence to algorithms and hence the outcome of resuscitation care [22, 65].

As a result, new resuscitation strategies relying on information technologies and devices aiming at improving non-technical skills and ensuring adherence to resuscitation guidelines at the team level are of interest. A previous pilot study observed better teamwork and communication using a situation display during simulated resuscitation scenarios [25]. However, there were no overall differences in SA scores between scenarios with and without the situation display as measured by the SAGAT. While the study was not a randomized controlled trial, it provided encouraging results regarding the impact of technology-based cognitive aids in achieving better non-technical skills among emergency department teams in the resuscitation event. In another study, Fitzgerald et al. found that a computer-aided decision support for experienced trauma teams on a large display resulted in improved protocol compliance and a significant reduction in error rates [66]. Unfortunately, the authors did not assess non-technical skills, which is of main interest to our study. Moreover, few studies have investigated the relationship between technical and non-technical skills on CPR performance [12, 67]. Our trial aims to address these flaws by measuring both technical and non-technical skills at the individual and team levels with a cluster randomized controlled trial approach during highly standardized simulation-based resuscitation scenarios.

Our study has some limitations. First, it will be conducted during a resuscitation simulation-based scenario, rather than tested in real-life situations. However, high-fidelity simulation is an essential investigative methodology to answer research questions that cannot be otherwise answered during real CPR as the diversity of patients and their diseases make such studies hard to standardize in critical situations [43]. Given that most results obtained from simulation-based resuscitation studies agree with those obtained from studies in real life, we are confident that InterFACE could be of great interest for real-life situations. Second, standardizing the scenario and the environment will help to avoid effect modifiers by limiting the influence of undesired variables on the outcomes. Third, the 15-min app training will be dispensed just prior to the scenario. In real life, the interval between training and actual use would probably be months. However, training with the app months before the study would unblind participants to its purpose and create a preparation bias. Finally, the Likert-type questionnaire that will be used to measure both perceived stress and satisfaction has not been assessed for validity, internal consistency, reliability, or generalizability. Although it cannot objectively measure these variables, it can be used to measure the difference of perceived stress and satisfaction.

To the best of our knowledge, InterFACE is the first shared decision-making mHealth tool aiming to improve team SA, communication, task performance, and leadership in real-time during in-hospital pediatric trauma and CPR. Importantly, such a digital tool has the potential to enhance adherence to PALS and ATLS algorithms and hence the outcome of resuscitation care. As research in this area is scarce, a study assessing its impact in a simulated environment is warranted before being able to assess it in real life-threatening situations in the vulnerable pediatric population.

## Trial status

The protocol version is 1.0 (13 March 2020). The trial has a declaration of no objection by Swissethics (Req-2020-00294). The trial is registered at ClinicalTrials.gov, ID: NCT04464603, 8 July 2020, https://clinicaltrials.gov/ct2/show/NCT04464603. The recruitment of subjects is expected to start late 2020. We anticipate that the intervention will be completed in early 2021 and study results will be available in mid 2021 (publication expected in mid-2021).

## Supplementary Information

**Additional file 1.** Standard Protocol Items: Recommendations for Interventional Trials (SPIRIT) 2013 Checklist [35].

**Additional file 2.** Goal-directed task analysis (GDTA) methodology. Adapted from [21]. The GDTA is a form of cognitive task analysis that hierarchically delineates decision-makers’ goals identified by experts in the domain, what critical decisions must be made in order to accomplish each goal, and the optimal information needed to make each decision as a basis for defining appropriate content for the development of SA assessment measures. Once completed, a composite “tree” is constructed for each simulation scenario. SA requirements identified through the GDTA can be used to create objective metrics for evaluating the degree to which technologies are successful in supporting the SA of decision-makers.

**Additional file 3.** Detailed items of the situational awareness global assessment technique (SAGAT).

**Additional file 4.** Detailed items of the resuscitation team leader evaluation (RTLE) [48].

**Additional file 5.** Detailed items of the team emergency assessment measure (TEAM) [49].

**Additional file 6.** Consent form.

**Additional file 7.** Trial information summary according to the WHO Trial Registration Data Set (Version 1.3.1).

## Data Availability

The final datasets established and analyzed will not be publicly available. An anonymous copy of the final datasets underlying publications resulting from this study will be available from the corresponding author upon reasonable and approved request.

## Abbreviations

AHA: American Heart Association
ATLS: Advanced trauma life support
CA: Cardiac arrest
CI: Confidence interval
CONSORT: Consolidated standards of reporting trials
CPR: Cardiopulmonary resuscitation
GDTA: Goal-directed task analysis
InterFACE: Interconnected and focused mobile apps on patients’ care environment
LCD: Liquid crystal display
N/A: Not applicable
PALS: Pediatric advanced life support
PED: Pediatric emergency department
PedAMINES: Pediatric accurate medication in emergency situations
p-IHCA: Pediatric in-hospital cardiac arrest
p-OHCA: Pediatric out-of-hospital cardiac arrest
ROSC: Return of spontaneous circulation
RTLE: Resuscitation team leader evaluation
SA: Situational awareness
SD: Standard deviation
SPIRIT: Standard Protocol Items: Recommendations for Interventional Trials
SUS: System usability scale
TEAM: Team emergency assessment measure
(T)SAGAT: (Team) situational awareness global assessment technique
pVT: Pulseless ventricular tachycardia
