# Supplementary figures and images for: Impact of a shared decision-making mHealth tool on caregivers’ team situational awareness, communication effectiveness, and performance during pediatric cardiopulmonary resuscitation: study protocol of a cluster randomized controlled trial
Source: Trials. 2021 Apr 13;22:277. doi: 10.1186/s13063-021-05170-3 (PMC8042906; doi:10.1186/s13063-021-05170-3)

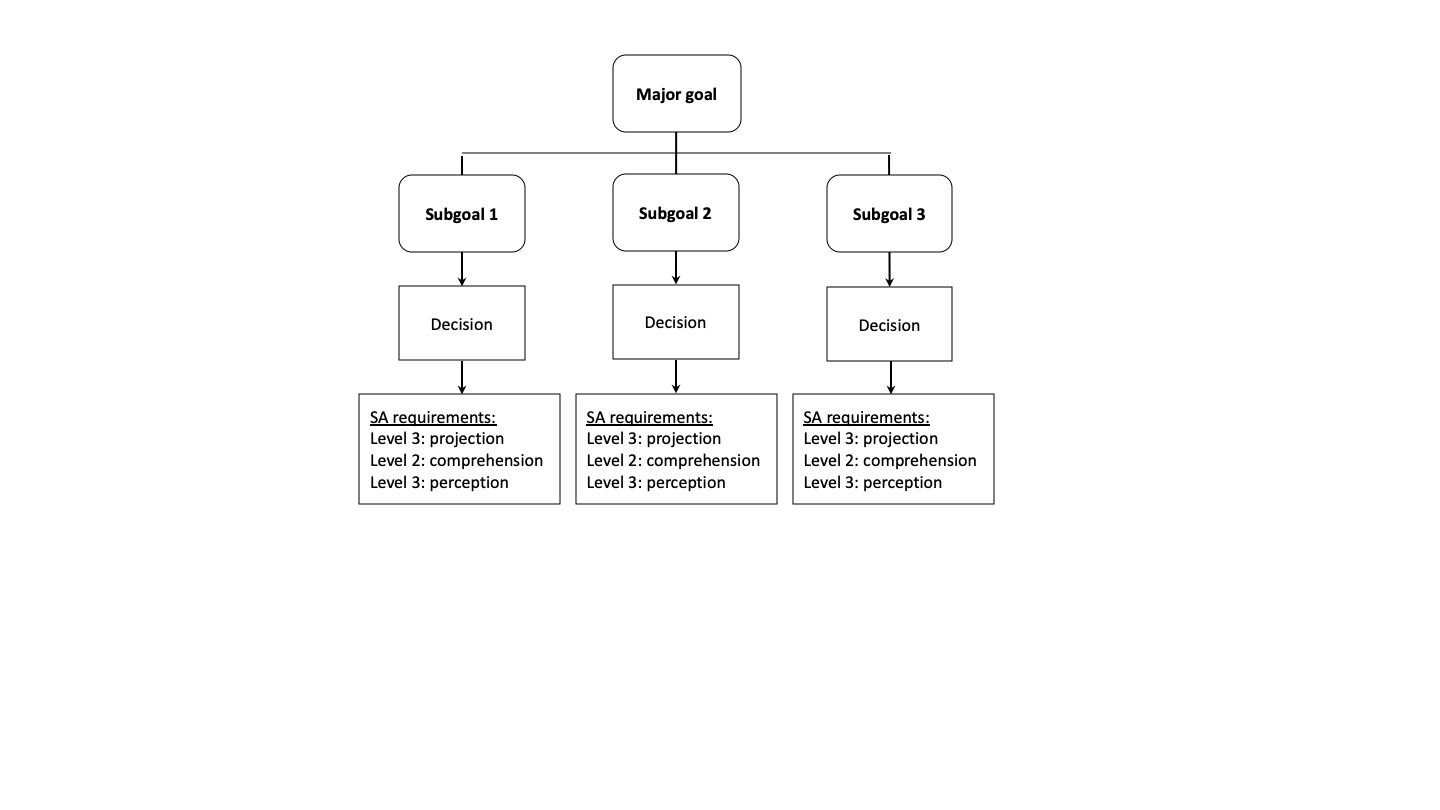

Supplement: Supplementary file 2 — Additional file 2. Goal-directed task analysis (GDTA) methodology. Adapted from [21]. The GDTA is a form of cognitive task analysis that hierarchically delineates decision-makers’ goals identified by experts in the domain, what critical decisions must be made in order to accomplish each goal, and the optimal information needed to make each decision as a basis for defining appropriate content for the development of SA assessment measures. Once completed, a composite “tree” is constructed for each simulation scenario. SA requirements identified through the GDTA can be used to create objective metrics for evaluating the degree to which technologies are successful in supporting the SA of decision-makers. [file 13063_2021_5170_MOESM2_ESM.png]

**Additional file 4.**


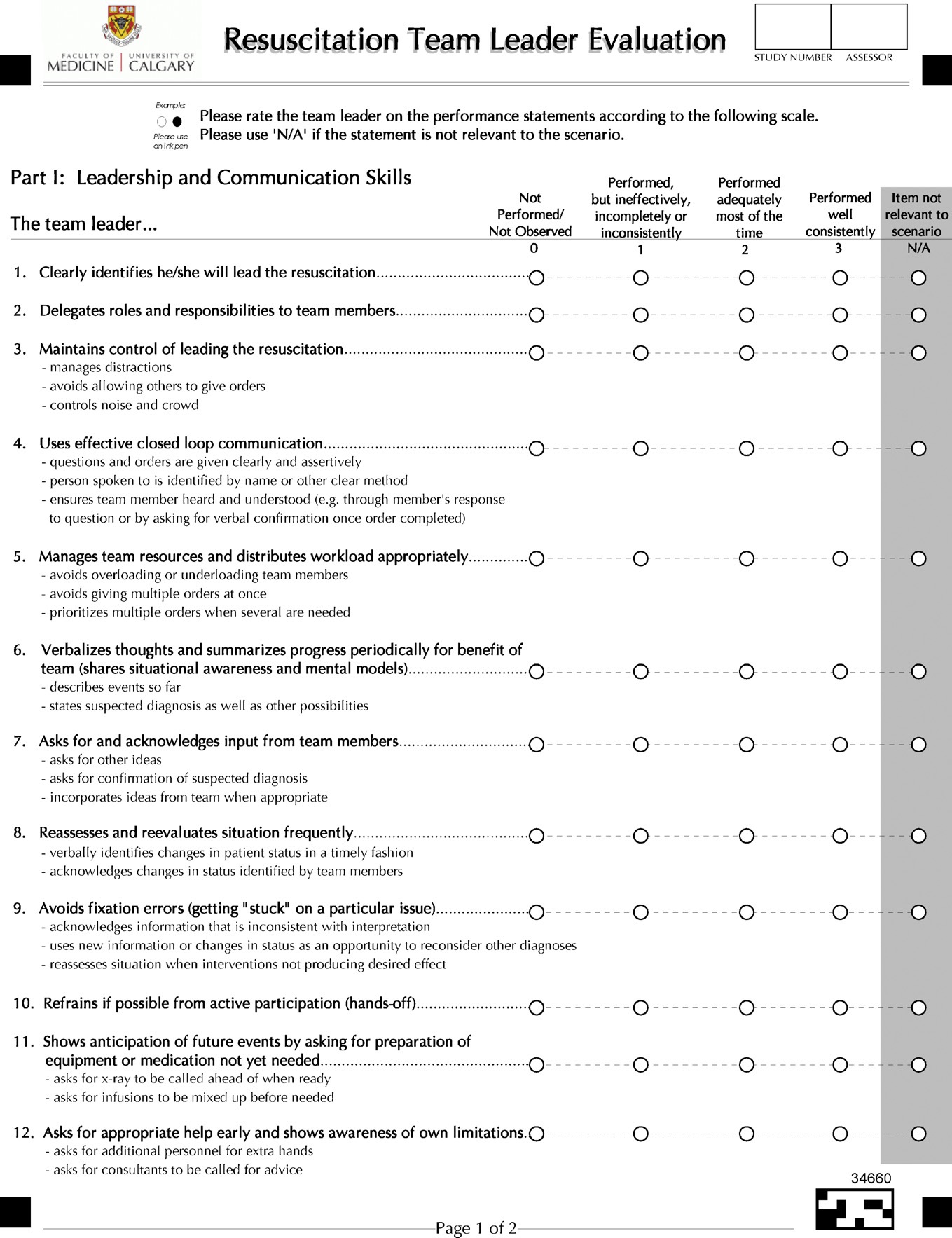

Supplement: Supplementary file 4 — Additional file 4. Detailed items of the resuscitation team leader evaluation (RTLE) [48]. [file 13063_2021_5170_MOESM4_ESM.docx]

**Additional file 5.**


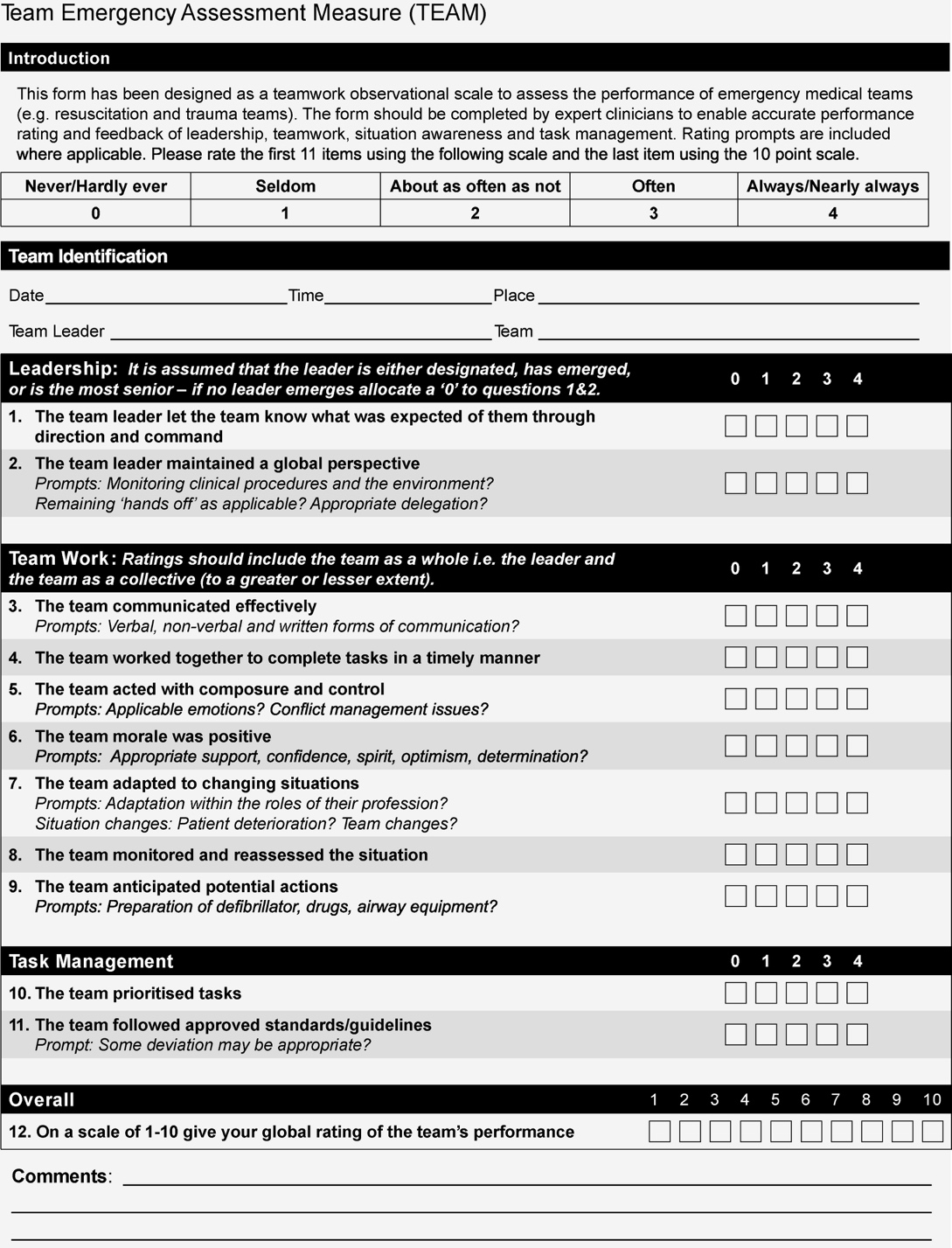

Supplement: Supplementary file 5 — Additional file 5. Detailed items of the team emergency assessment measure (TEAM) [49]. [file 13063_2021_5170_MOESM5_ESM.docx]
