# Supplementary material for: Impact of a shared decision-making mHealth tool on caregivers’ team situational awareness, communication effectiveness, and performance during pediatric cardiopulmonary resuscitation: study protocol of a cluster randomized controlled trial
Source: Trials. 2021 Apr 13;22:277. doi: 10.1186/s13063-021-05170-3 (PMC8042906; doi:10.1186/s13063-021-05170-3)
Supplement: Supplementary file 3 — Additional file 3. Detailed items of the situational awareness global assessment technique (SAGAT). [file 13063_2021_5170_MOESM3_ESM.docx]

**SAGAT participant query form scenario 1**

**Level 1 (*perception*)**

1. Please indicate on the figure below and next to each pictured individual, the letter corresponding to the role played by that team member:


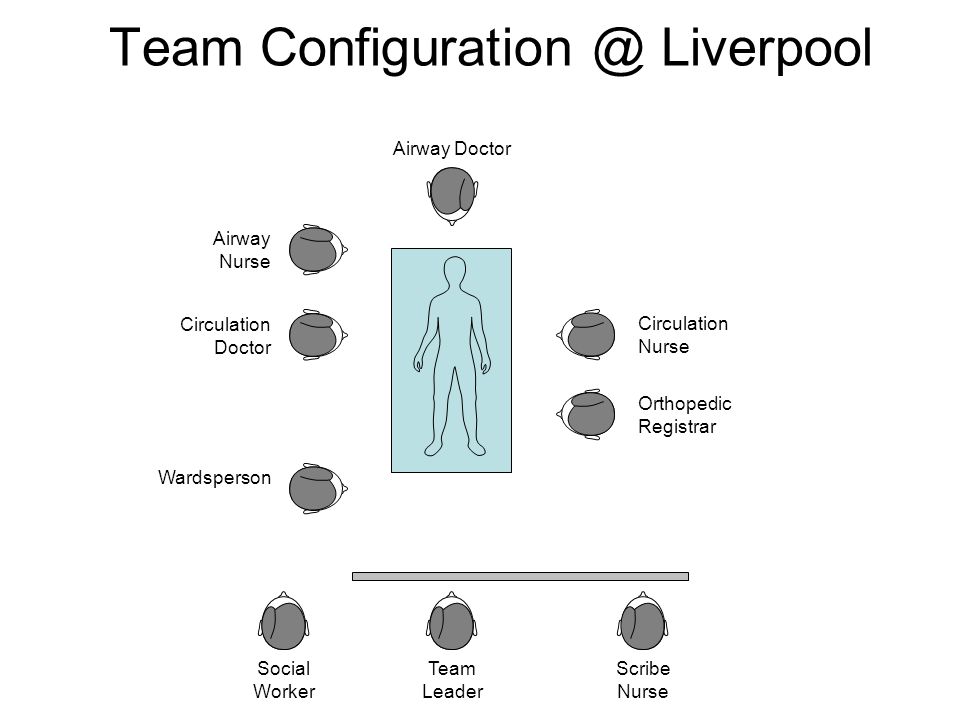

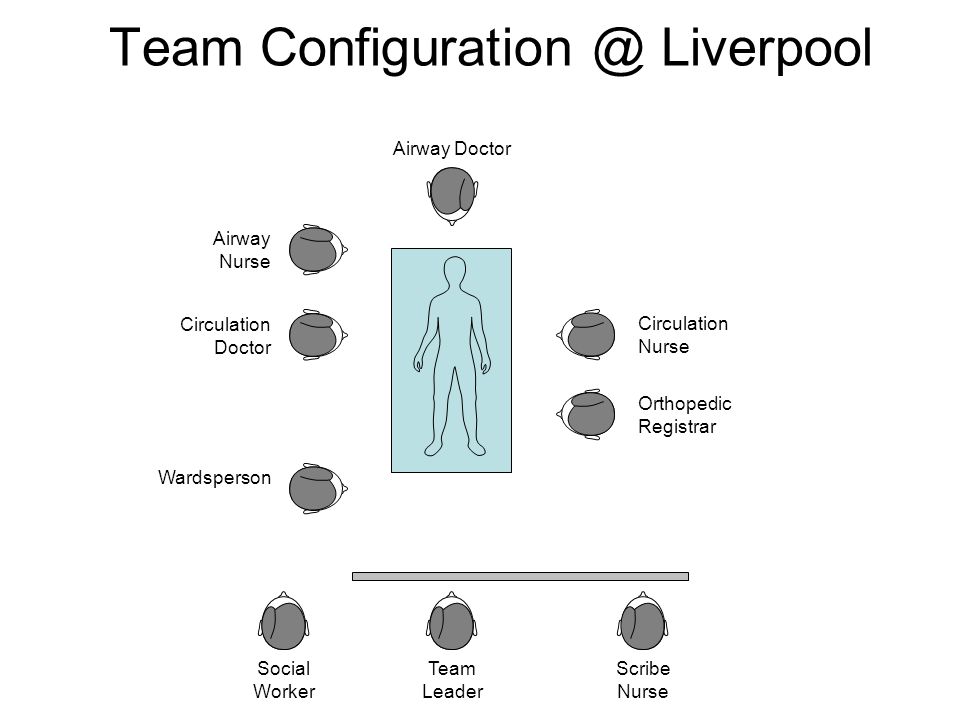

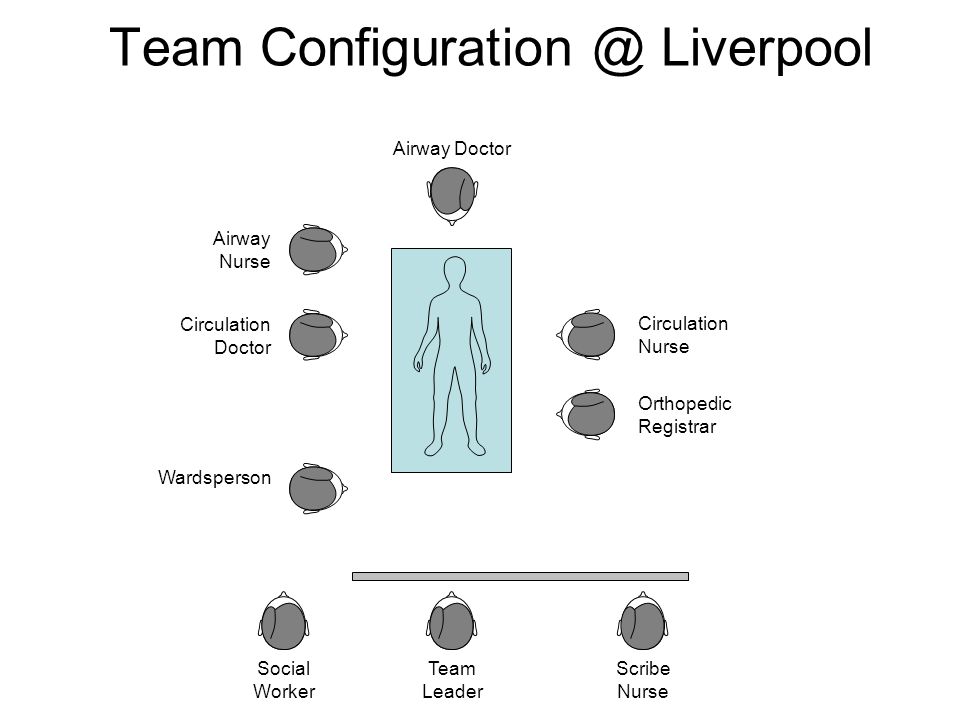

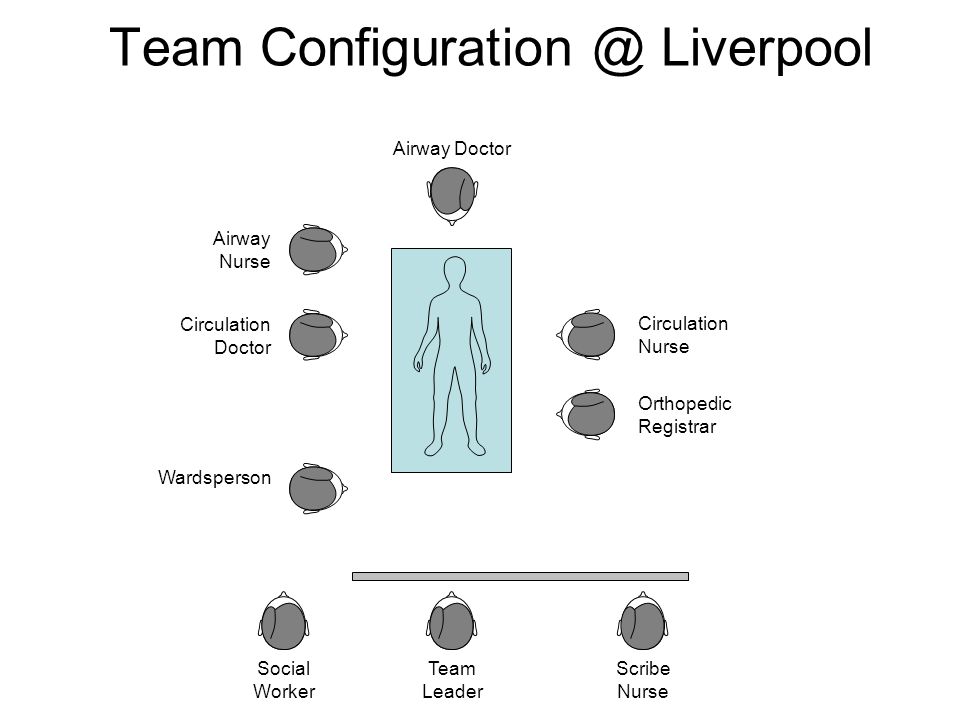

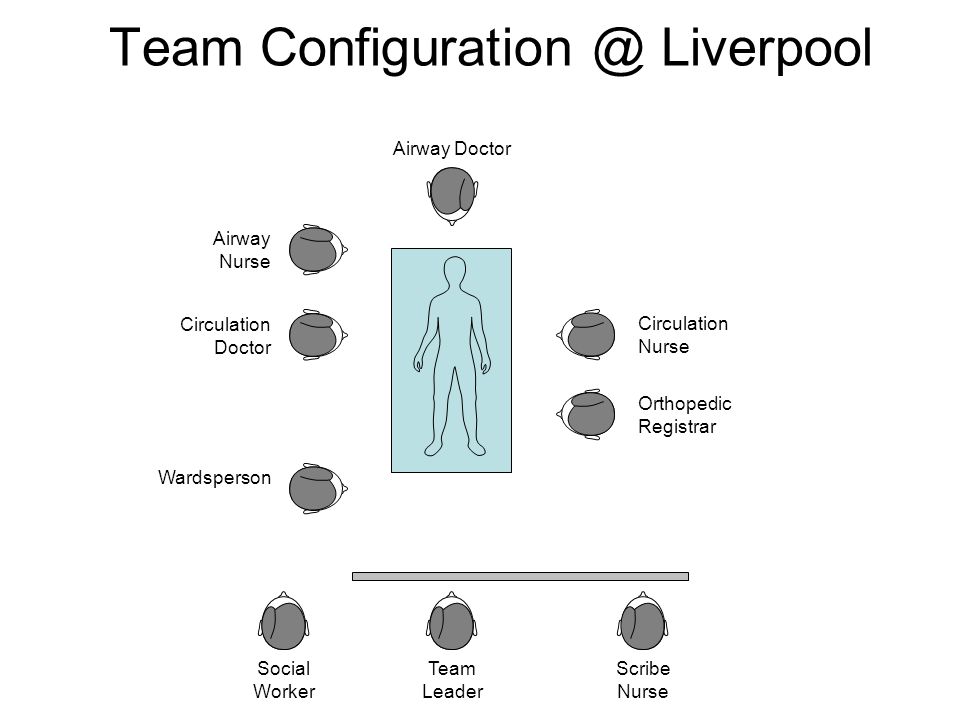

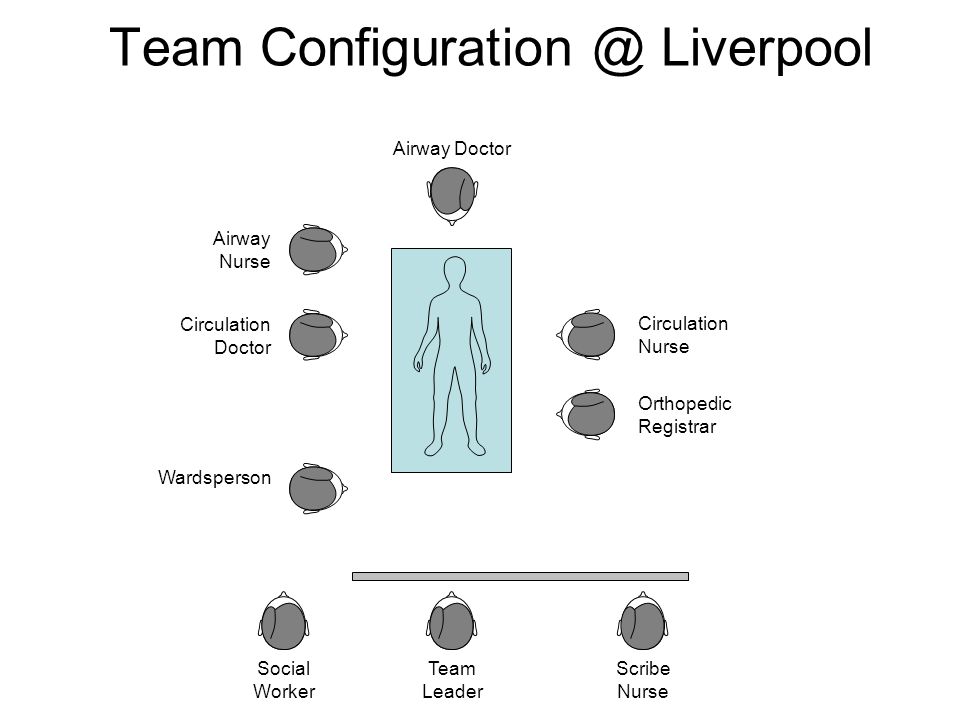

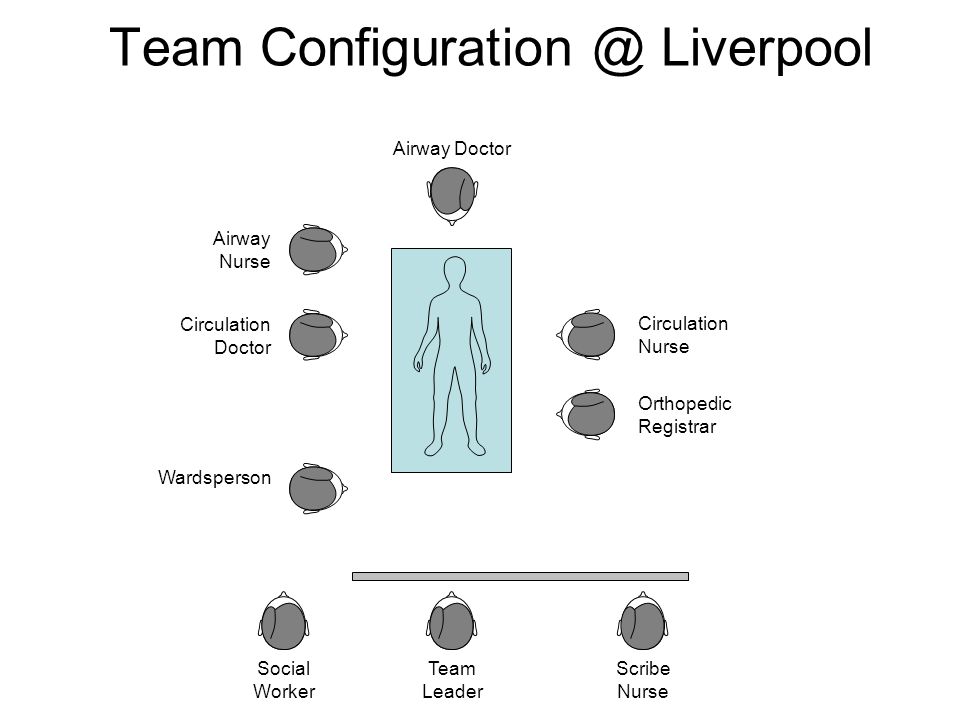

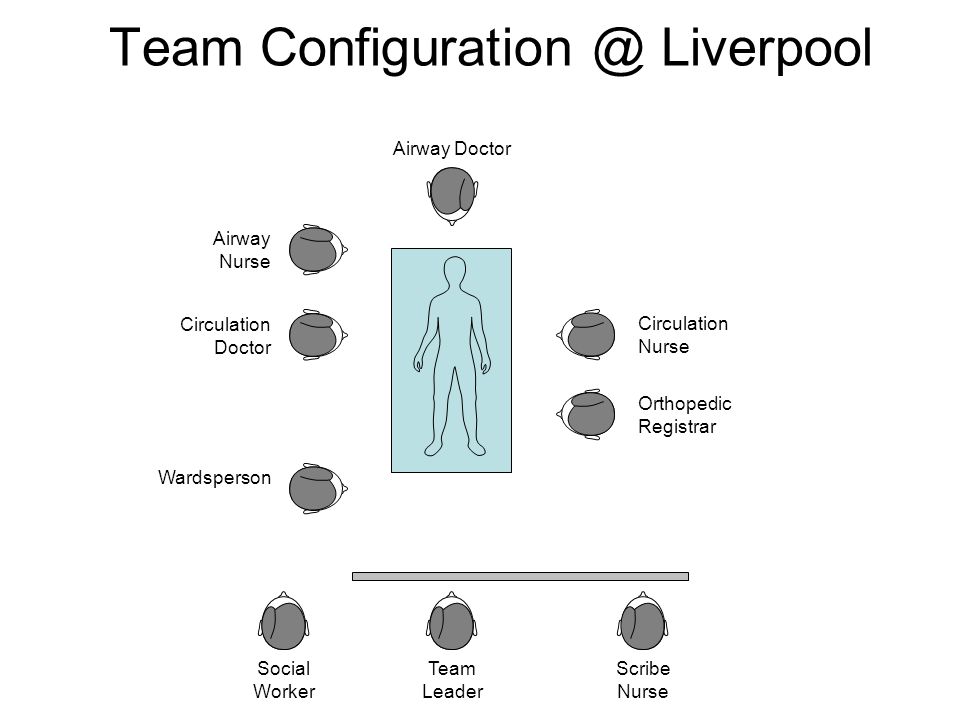

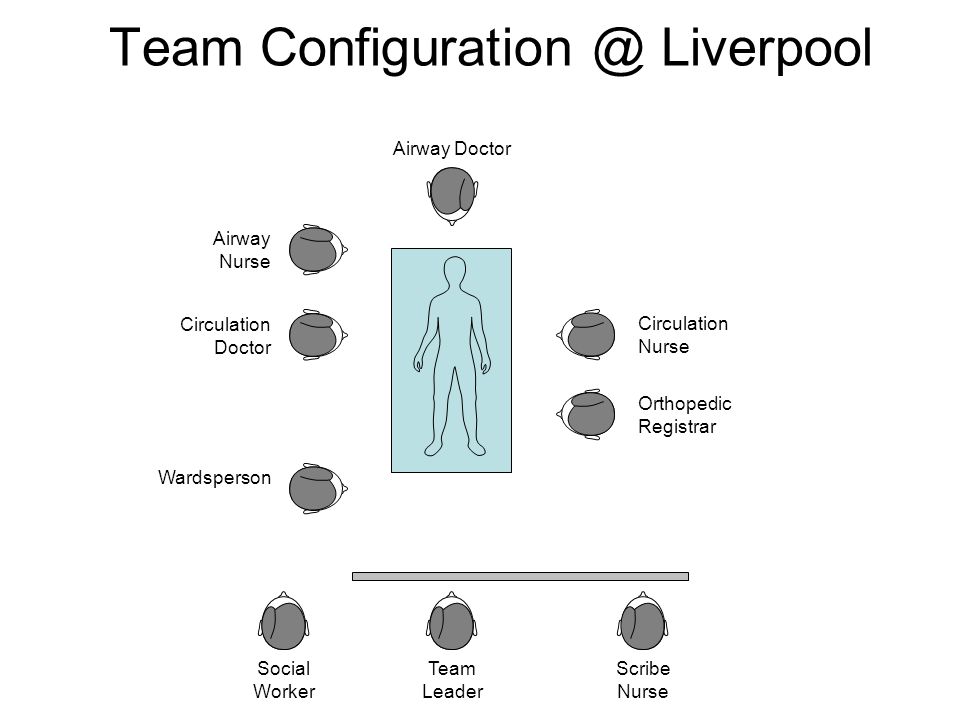

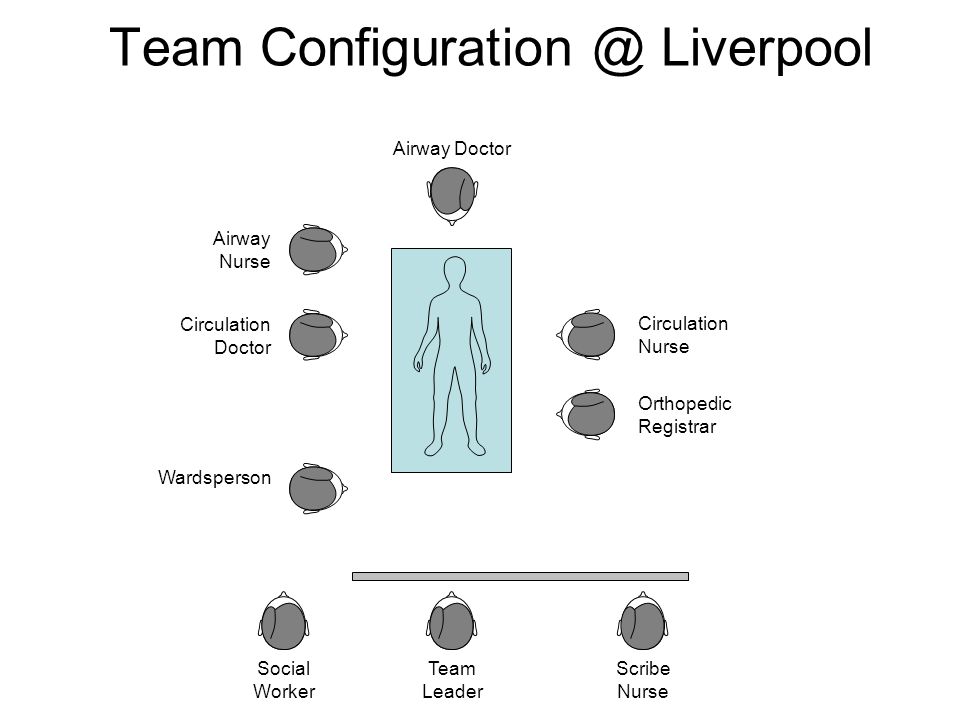

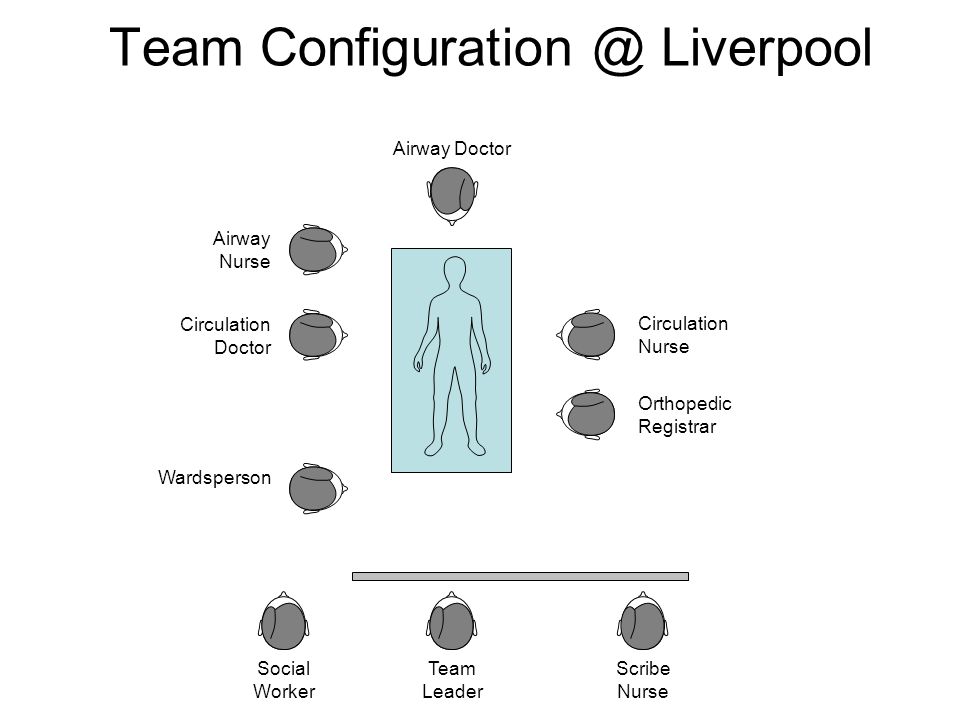


1. Physician 1 - assessment
2. Physician 2 – team leader
3. Airway specialist / Anesthetist
4. Nurse 1 – monitoring and procedures
5. Nurse 2 – circulation and drug preparation
6. Nurse 3 - scribe
7. Please state your assessment of the patient’s airway patency:
8. Opened
9. Threatened
10. Obstructed
11. I don’t know
12. Please state your assessment of the patient’s work of breathing:
13. Normal work of breathing
14. Increased work of breathing
15. Respiratory failure
16. I don’t know
17. Please state your assessment of the last displayed oxygen saturation:
18. >94%
19. 90-94%
20. <90 %
21. Not available
22. I don’t know
23. Please state your assessment of the patient’s circulation:
24. Normal circulation
25. Decreased circulation, without circulatory failure
26. Circulatory failure
27. I don’t know
28. Please indicate the last displayed heart rate of the patient:
29. 0/min
30. <60/min
31. 60-100/min
32. >100/min
33. I don’t know
34. Please indicate the systolic blood pressure of the patient:
35. 0 mmHg
36. > 0 but < 70 mmHg
37. > 70 mmHg
38. I don’t know
39. Please state your assessment of the patient’s consciousness
40. Alert
41. Responsive to verbal stimuli
42. Responsive to pain
43. Unresponsive
44. I don’t know
45. Please state your assessment of the patient’s pupils:
46. Constricted
47. Normal, responsive to light
48. Unilaterally dilated
49. I don’t know
50. What was the last manual defibrillator setting?
51. Defibrillation mode, energy dose 6-12 Joules
52. Defibrillation mode, energy dose 24 Joules
53. Defibrillation mode, energy dose 48 Joules
54. Synchronized cardioversion mode, energy dose 6-12 Joules
55. Transcutaneous pacing mode, pacing rate 100/min
56. What is the patient’s heart rhythm?
57. Sinusal
58. Asystole
59. Pulseless electrical activity (PEA)
60. Pulseless ventricular tachycardia (pVT)
61. Ventricular fibrillation (FV)
62. I don’t know
63. How many times has the patient been defibrillated so far?
64. 0
65. 1
66. 2
67. 3
68. 4
69. I don’t know
70. How many times has the patient received epinephrine so far?
71. 0
72. 1
73. 2
74. 3
75. 4
76. I don’t know
77. Which drug has been delivered to the patient so far?
78. epinephrine
79. amiodarone
80. epinephrine and amiodarone
81. adenosine
82. none of the above
83. I don’t know
84. How many Joules have been delivered to the patient for the last defibrillation?
85. 6
86. 12 J
87. 24 J
88. 48 J
89. > 48 J
90. I don’t know
91. Has help from a specialist been discussed and requested?
92. yes
93. no
94. I don’t know
95. What is the medical equipment already in place on the patient?
96. Monitoring
97. Monitoring + IV/IO access
98. Monitoring + IV/IO access + defibrillator patches
99. Monitoring + IV/IO access + defibrillator patches + ECG leads
100. Monitoring + IV/IO access + defibrillator patches + ECG leads + C-spine protection
101. I don’t know
102. What is the patient’s last measured temperature?
103. < 36°C
104. 36 - 36.5°C
105. 36.5 - 37.0°C
106. 37.0 - 37.5°C
107. > 37.5°C
108. I don’t know

19. Which part of the primary survey was the last one to be evaluated?

1. Airways (A)
2. Breathing (B)
3. Circulation (C)
4. Disability (D)
5. Exposure (E)
6. I don’t know

20. Which PALS algorithm is currently used by your team?

1. Bradycardia
2. Tachycardia with a pulse and adequate perfusion
3. Tachycardia with a pulse and poor perfusion
4. Cardiac arrest
5. I don’t know

21. Which cardiac arrest algorithm is currently used by your team?

1. Asystole
2. Pulseless electrical activity (PEA)
3. Pulseless ventricular tachycardia (pVT)
4. Ventricular fibrillation (FV)
5. I don’t know

22. How long has it been since CPR was initiated?

1. < 5 min
2. 5 – 10 min
3. 11 – 15 min
4. > 15 min
5. I don’t know

23. Is there any CPR algorithm projected on the screen?

1. Yes
2. No

**Level 2 (*comprehension*)**

1. What is the primary problem of the patient?

1. Airway
2. Breathing
3. Circulation
4. Disability
5. Rhythm disturbances
6. A combination of 2 or more of the above-mentioned
7. I don’t know

2. Please state your diagnosis (most urgent problem to treat for this patient)?

1. Asystole
2. Sepsis
3. Hypovolemic shock due to a hemorrhage
4. pVT
5. Major brain injury with delayed loss of consciousness
6. VF
7. SVT with poor perfusion
8. Intoxication
9. Suspected parental neglect
10. I don’t know

3. Is the patient adequately oxygenated?

1. Yes
2. No
3. I don’t know
4. What is the cause of the respiratory failure?
5. Upper airway obstruction
6. Lower airway obstruction
7. Lung parenchymal disease
8. Disordered control of breathing
9. I don’t know

5. What is the hemodynamic status of the patient?

1. Normal hemodynamic parameters
2. Compensated shock
3. Hypotensive shock
4. I don’t know

6. What is the respiratory status of the patient?

1. Normal
2. Respiratory distress
3. Respiratory failure
4. Apneic
5. I don’t know
6. What kind of shock does the patient suffer from?
7. The patient is not in shock
8. Hypovolemic shock
9. Distributive shock
10. Cardiogenic shock
11. Obstructive shock
12. I don’t know

8. What is the current trajectory of the patient?

1. No change
2. Improving
3. Getting worse
4. I don’t know

**Level 3 (*projection*)**

1. Who is the team leader at this point?

2. Which action needs to be prioritized at this point?

1. To deliver a shock
2. To administer a drug
3. To ensure post-resuscitation (ROSC) care
4. To call the cardiologist
5. I don’t know

3. Which drugs are required to treat the patient’s current most urgent issue?

1. Adenosine 0.1 mg/kg rapid IV bolus and amiodarone 5 mg/kg
2. Epinephrine 0.1 mL/kg (0.1 mg/ml concentration) and amiodarone 5 mg/kg
3. Epinephrine 0.1 mL/kg (0.1 mg/ml concentration) and atropine 0.02 mg/kg
4. Adenosine 0.1 mg/kg rapid IV bolus and atropine 0.02 mg/kg
5. I don’t know

4. Is the team complete at this point or do any other health care professionals still need to be notified? If so, which professional needs to be included in the team?

1. Pneumologist
2. Anesthetist
3. Pediatric intensive care unit physician
4. None
5. I don’t know

5. If the patient’s condition does not improve, what is susceptible to happen to the heart rhythm?

1. To progress toward asystole
2. To progress toward PEA
3. To progress toward pVT
4. To progress toward SVT with poor perfusion
5. To progress toward VF
6. To progress toward sinus rhythm
7. I don’t know

6. If the condition does not improve, what is susceptible to happen to the blood pressure?

1. No change
2. To increase continuously
3. To decrease continuously
4. To fluctuate
5. I don’t know

7. What medication may still be required at the next step?

1. Epinephrine
2. Amiodarone
3. Succinylcholine
4. Atropine
5. Ketamine
6. I don’t know

8. Please state your assessment of overall team function (1 [very poor] to 10 [excellent teamwork]

1 - 2 - 3 - 4 - 5 - 6 - 7 - 8 - 9 - 10

**SAGAT participant query form scenario 2**

**Level 1 (*perception*)**

1. Please indicate on the figure below and next to each pictured individual, the letter corresponding to the role played by that team member:


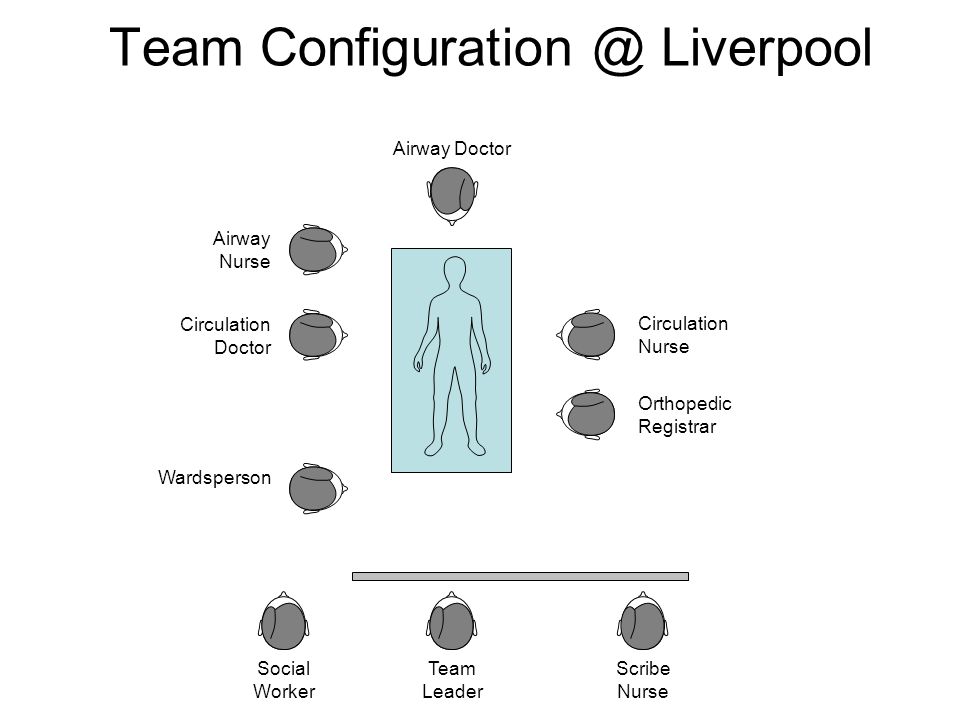

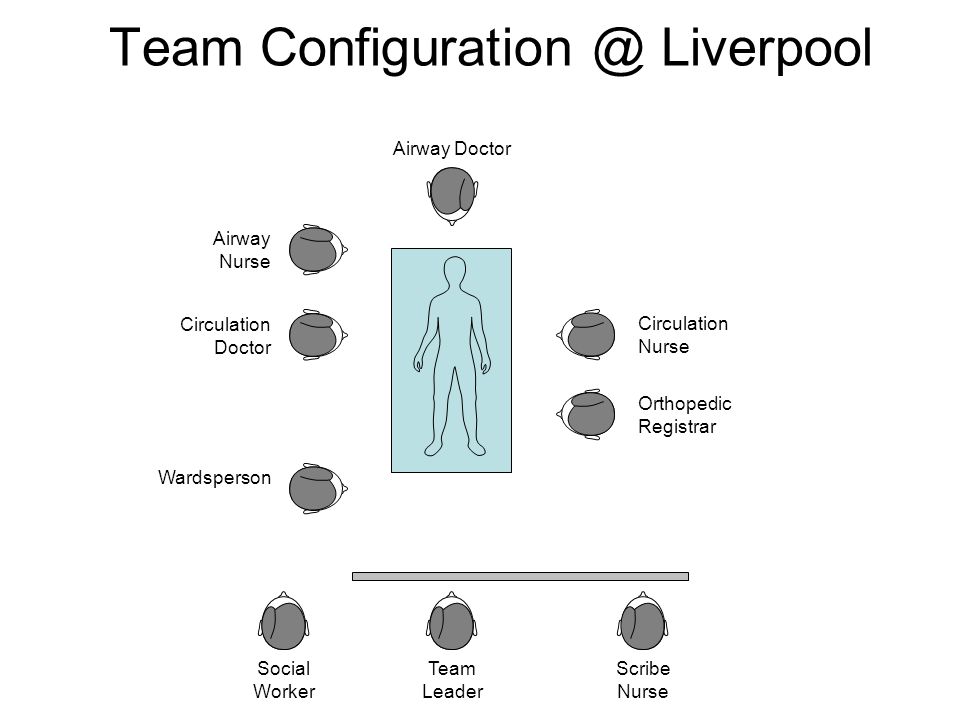

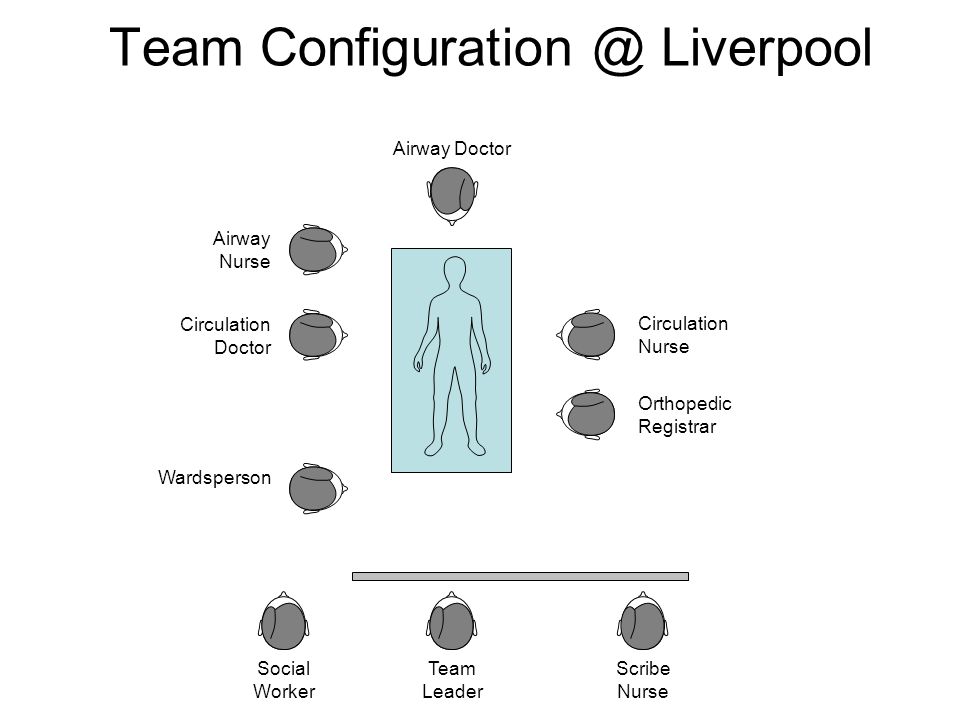

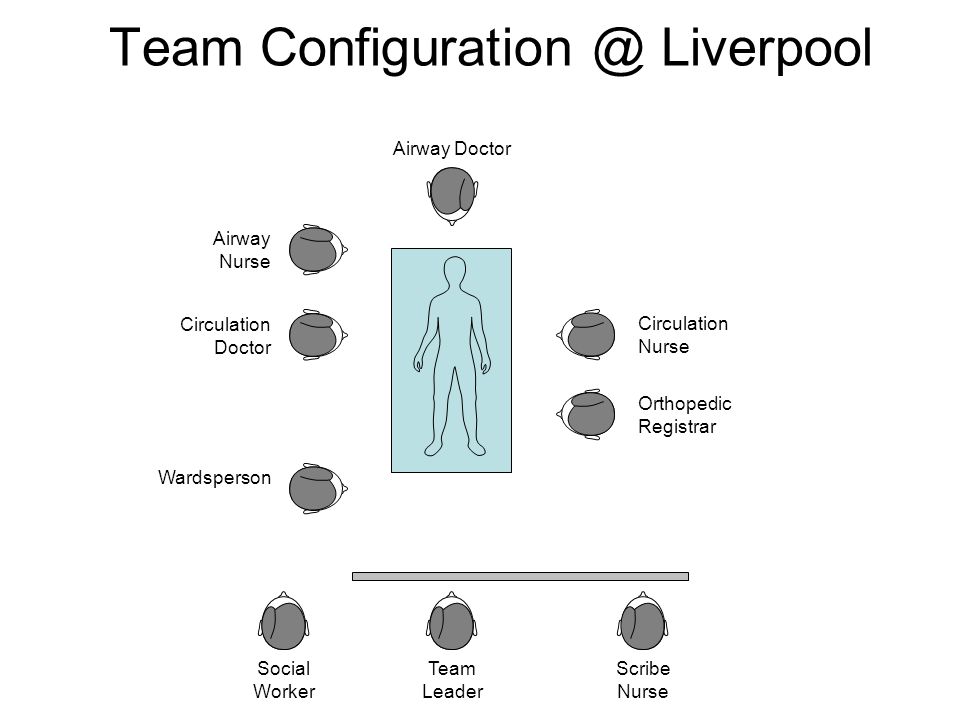

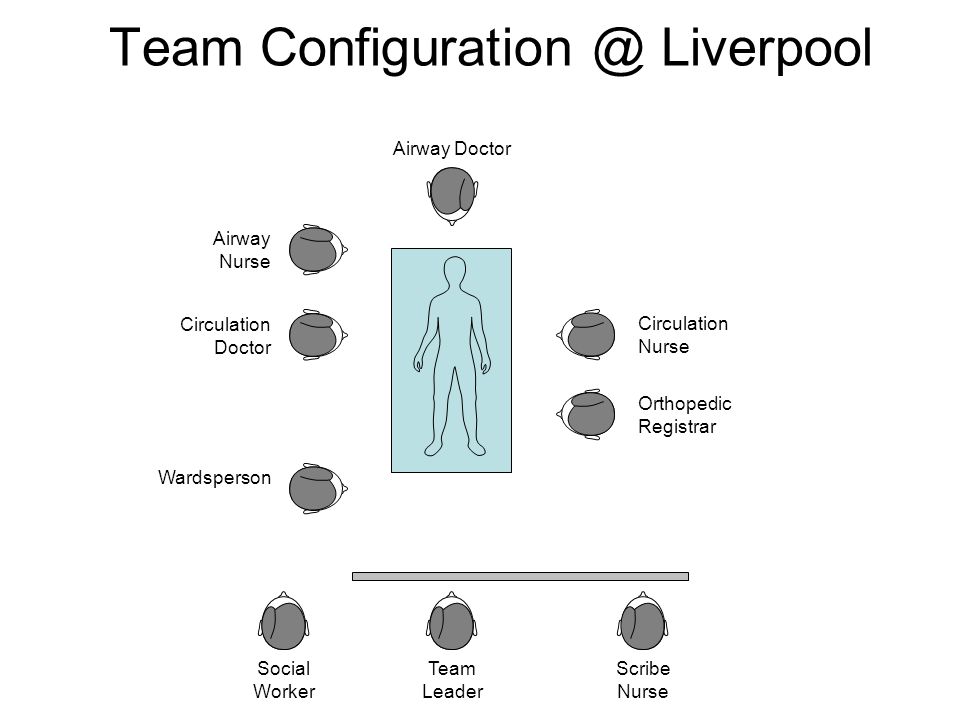

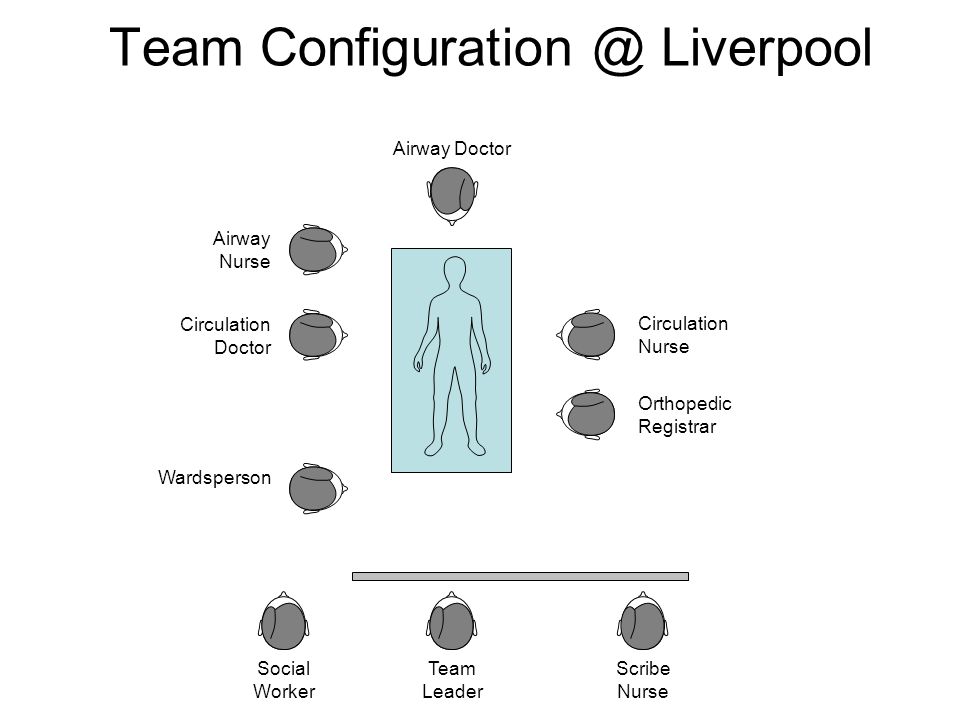

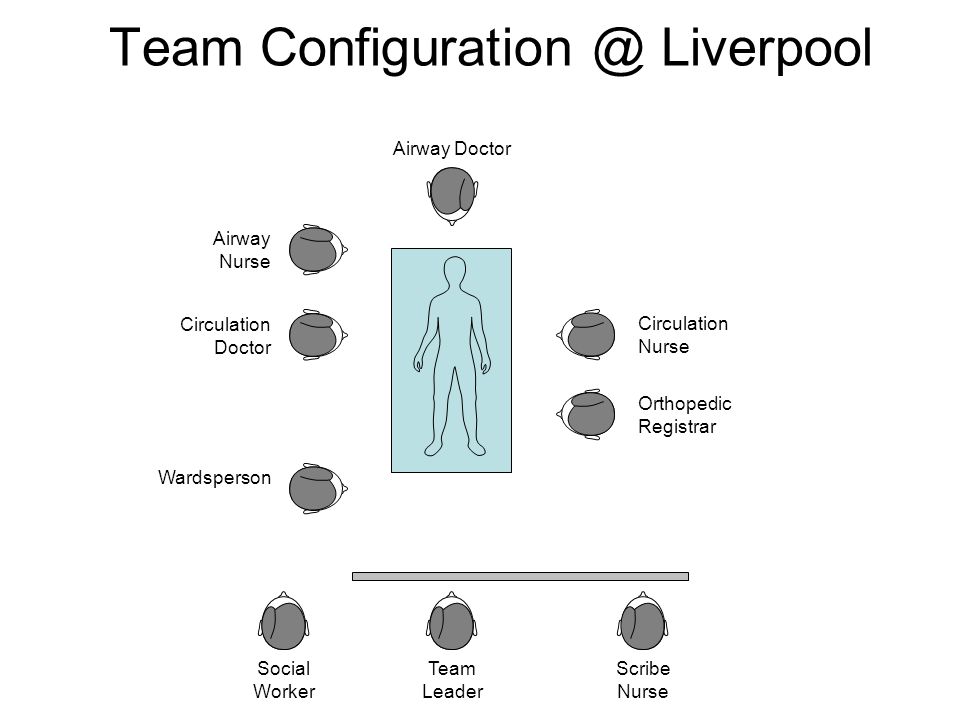

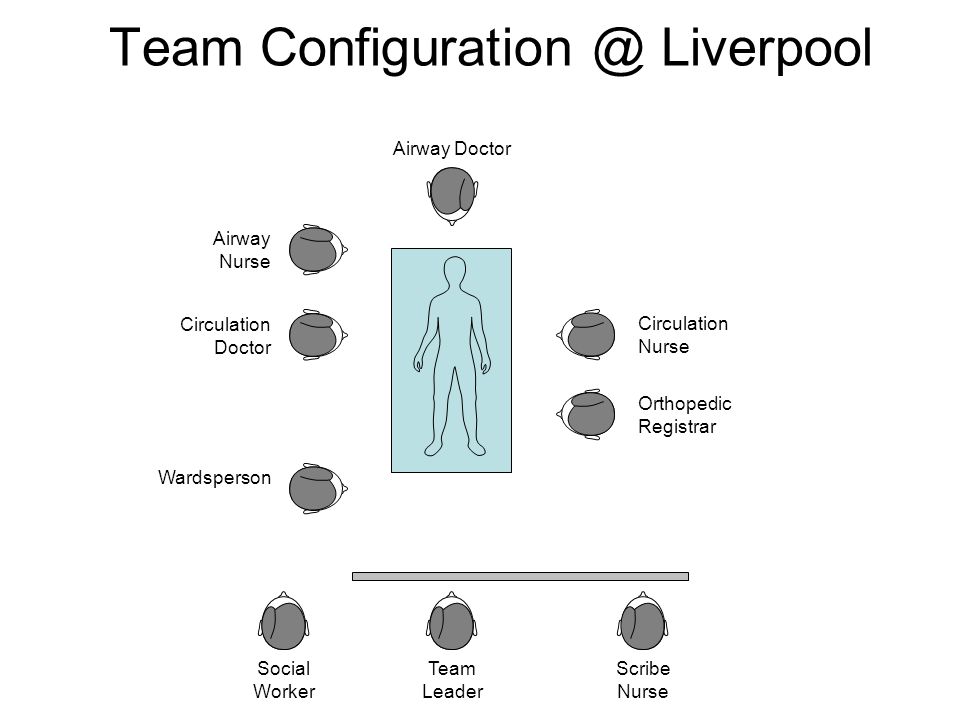

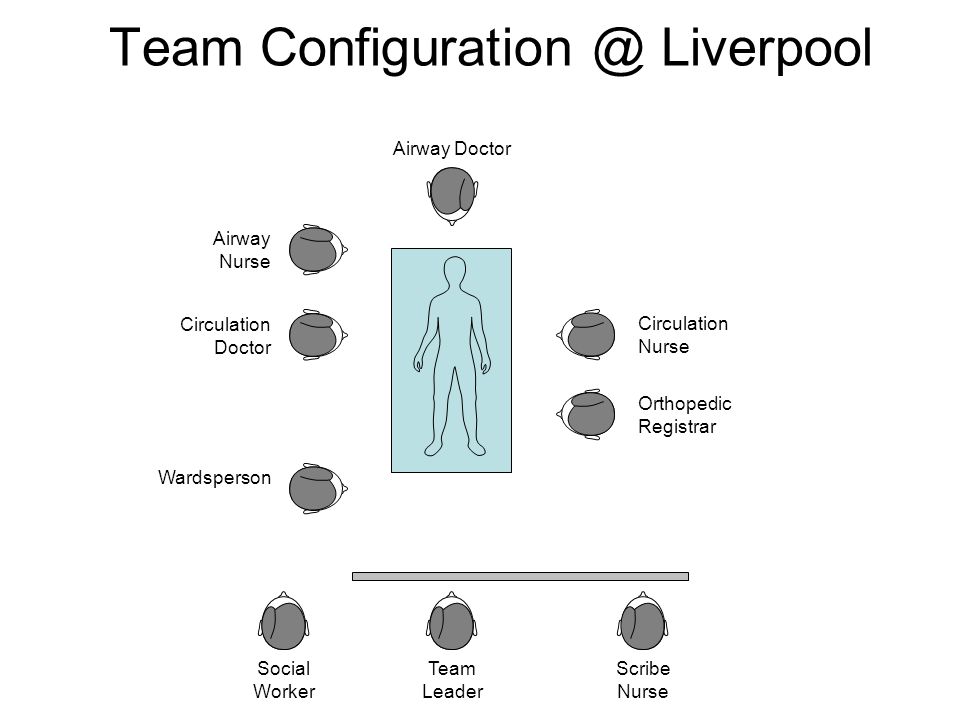

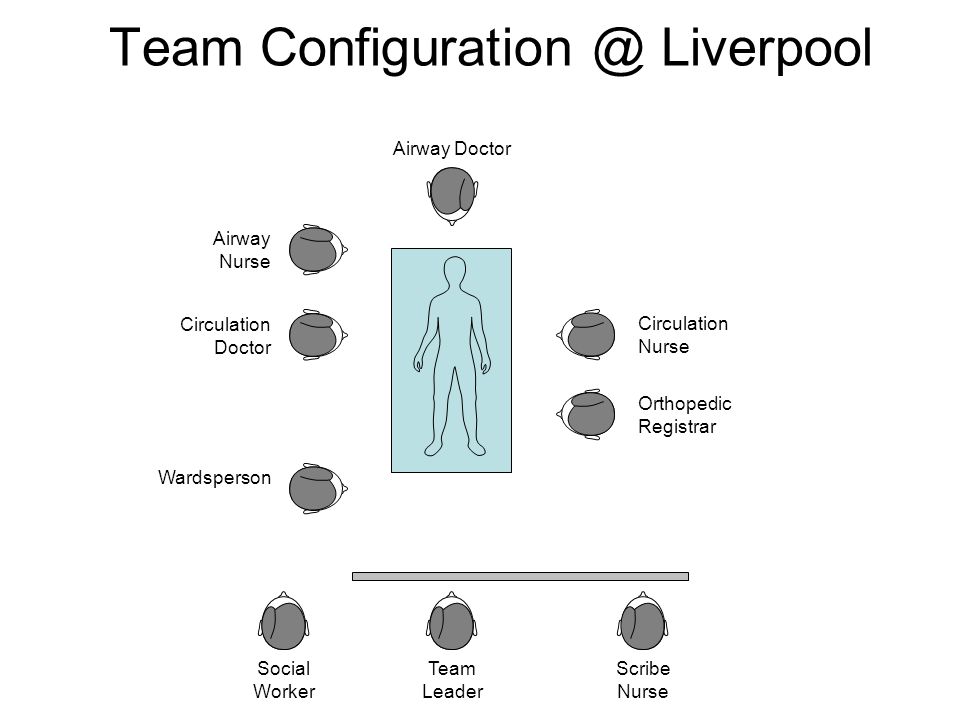

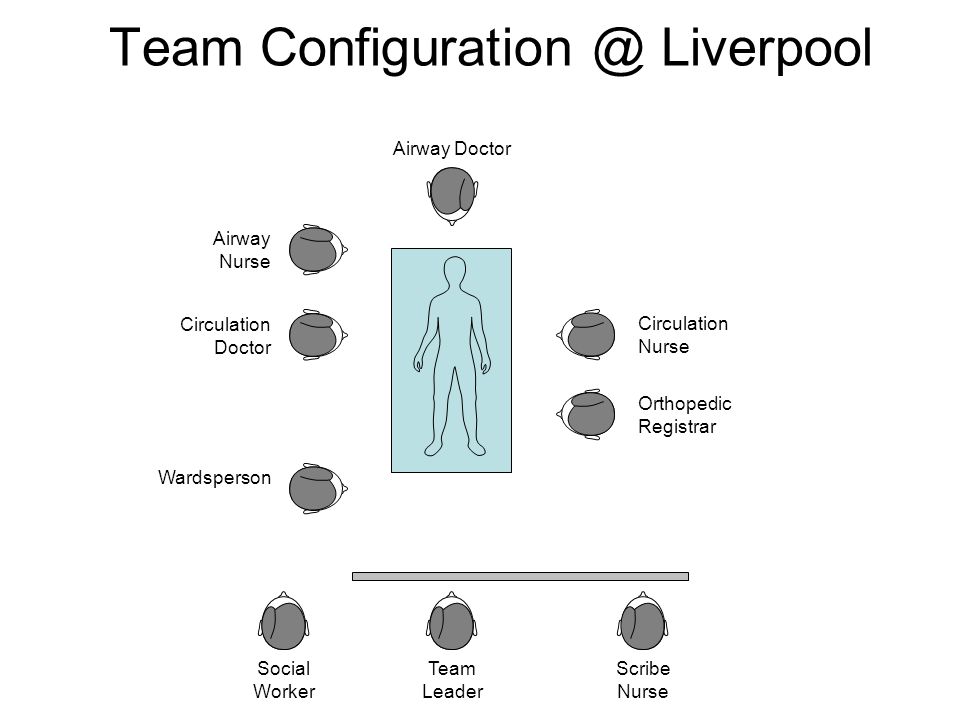


1. Physician 1 - assessment
2. Physician 2 – team leader
3. Airway specialist / Anesthetist
4. Nurse 1 – monitoring and procedures
5. Nurse 2 – circulation and drug preparation
6. Nurse 3 - scribe
7. Please state your assessment of the patient’s airway patency:
8. Opened
9. Threatened
10. Obstructed
11. I don’t know
12. Please state your assessment of the patient’s spine immobilization:
13. C-spine secured by a cervical collar
14. C-spine NOT neither secured by a cervical collar nor manually
15. C-spine manually secured by maintaining the head aligned
16. I don’t know
17. Please state your assessment of the last displayed oxygen saturation:
18. >94%
19. 90-94%
20. <90
21. Not available
22. I don’t know
23. Please state your assessment of the patient’s circulation:
24. Normal circulation
25. Decreased circulation, without circulatory failure
26. Circulatory failure
27. I don’t know
28. Please indicate the last displayed heart rate of the patient:
29. 0/min
30. <60/min
31. 60-100/min
32. >100/min
33. I don’t know
34. Please indicate the systolic blood pressure of the patient:
35. 0 mmHg
36. > 0 but < 70 mmHg
37. > 70 mmHg
38. I don’t know
39. Please state your assessment of this patient’s consciousness:
40. Alert
41. Responsive to verbal stimuli
42. Responsive to pain
43. Unresponsive
44. I don’t know
45. Please state your assessment of the patient’s pupils:
46. Constricted
47. Normal, responsive to light
48. Unilaterally dilated
49. I don’t know
50. Please state your assessment of the patient’s Glasgow coma scale:
51. 15/15
52. 9 to14/15
53. 4 to 8/15
54. 3/15
55. I don’t know
56. What is the current pulse rate range of the patient?
57. Normal range
58. Bradycardia
59. Tachycardia
60. No pulse
61. I do not know
62. How much intravenous crystalloids (in millilitres) has already been administered to the patient?
63. 10 ml/kg
64. 20 ml/kg
65. 40 ml/kg
66. 60 ml/kg
67. I don’t know
68. Has the patient received any blood transfusions?
69. yes
70. no
71. I don’t know
72. Has help from a specialist been discussed and requested?
73. yes
74. no
75. I don’t know
76. What is the medical equipment already in place on the patient?
77. Monitoring
78. Monitoring + IV/IO access
79. Monitoring + IV/IO access + C-spine protection
80. Monitoring + IV/IO access + C-spine protection+ pelvic binder
81. I don’t know
82. What is the patient’s last measured temperature?
83. < 36°C
84. 36 - 36.5°C
85. 36.5 - 37.0°C
86. 37.0 – 37.5°C
87. > 37.5°C
88. I don’t know

17. Which part of the primary survey was the last one to be evaluated?

1. Airways (A)
2. Breathing (B)
3. Circulation (C)
4. Disability (D)
5. Exposure (E)
6. I don’t know

18. What exam has already been ordered (multiple answer question)?

1. Blood typing and cross-match
2. Chest X-ray
3. Pelvic and femur X-rays
4. FAST
5. CT-scan
6. I don’t know

19. Which part of the body has been injured (multiple answer question)?

1. Head
2. Thorax
3. Abdomen
4. Limbs
5. Polytrauma (≥ 2 major systems)
6. I don’t know

20. How long has it been since CPR was initiated?

1. < 5 min
2. 5 – 10 min
3. 11 – 15 min
4. > 15 min
5. I don’t know

21. Has any bone fracture(s) already been noticed?

1. Yes: single, closed
2. Yes: single, open
3. Yes: multiple, open
4. Yes: multiple, closed
5. None seen or suspected
6. I don’t know

22. Is there any CPR algorithm projected on the wall?

1. Yes
2. No

**Level 2 (*comprehension*)**

1. What is the primary problem of the patient?

1. Airway
2. Breathing
3. Circulation
4. Disability
5. Rhythm disturbances
6. Some of the above combined
7. I don’t know

2. Please state your diagnosis (most urgent problem to treat for this patient)?

1. Asystole
2. Sepsis
3. Hypovolemic shock due to a hemorrhage
4. pVT
5. Major brain injury with delayed loss of consciousness
6. VF
7. SVT with poor perfusion
8. Intoxication
9. Suspected parental neglect
10. I don’t know

3. What is the current life-threatening issue in the patient?

1. Hemothorax
2. Tension pneumothorax
3. Abdominal/pelvic hemorrhage
4. Limb fracture with massive blood loss
5. Closed head injury
6. I don’t know

4. Is the patient adequately oxygenated?

1. Yes
2. No
3. I don’t know

5. Is fluid resuscitation sufficient?

1. Yes
2. No
3. I don’t know

6. What is the hemodynamic state of the patient?

1. Normal hemodynamic parameters
2. Compensated shock
3. Hypotensive shock
4. I don’t know

7. What kind of shock does the patient suffer from?

1. Not in shock
2. Hypovolemic shock
3. Distributive shock
4. Cardiogenic shock
5. Obstructive shock
6. I don’t know

8. What is the current trajectory of the patient?

1. No change
2. Improving
3. Getting worse
4. I don’t know

**Level 3 (*projection*)**

1. Who is the team leader at this point?

2. Which action needs to be prioritized at this point?

1. Mask/bag ventilation
2. Fluid resuscitation
3. CT-scan
4. Surgery
5. I don’t know

3. Is the team complete at this point or do any other health care professionals still need to be notified? If so, which specialist should be included in the resuscitation team in priority?

1. Trauma surgeon
2. Neurosurgeon
3. Pneumologist
4. Pediatric intensive care unit physician
5. None
6. I don’t know

4. If the condition does not improve, what will happen to the heart rate?

1. Increase
2. No change
3. Decrease
4. I don’t know

5. If the condition does not improve, what will happen to the blood pressure?

1. Increase
2. No change
3. Decrease
4. I don’t know

6. What investigations may be required?

1. Chest X-ray, facial X-ray and kidneys ultrasonography
2. Chest CT-scan and legs X-ray
3. Diagnostic peritoneal lavage
4. Abdominal ultrasonography, total body CT-scan
5. Hemoglobin measurement
6. I don’t know

7. What intervention may be required?

1. Hemothorax drainage
2. Crystalloids volume expansions, blood transfusion, surgery
3. 30-min electroencephalography recordings
4. Immediate transfer to the surgical unit for monitoring
5. Albumin infusion
6. I don’t know

8. Please state your assessment of overall team function (1 [very poor] to 10 [excellent teamwork])

1 - 2 - 3 - 4 - 5 - 6 - 7 - 8 - 9 - 10

*Adapted from M.S. Crozier et al [1] and 2018 ATLS 10th ed Student Course Manual [2].*

**References:**

1. Crozier MS, Ting HY, Boone DC, O'Regan NB, Bandrauk N, Furey A, et al. Use of human patient simulation and validation of the Team Situation Awareness Global Assessment Technique (TSAGAT): a multidisciplinary team assessment tool in trauma education. J Surg Educ. 2015;72(1):156-63. doi: 10.1016/j.jsurg.2014.07.009.

2. Henry S, Brasel K, Stewart RM. Student Course Manual Advanced Trauma Life Support ATLS. 10th ed: ACS American College of Surgeons; 2018.
