# Supplementary material for: Impact of a shared decision-making mHealth tool on caregivers’ team situational awareness, communication effectiveness, and performance during pediatric cardiopulmonary resuscitation: study protocol of a cluster randomized controlled trial
Source: Trials. 2021 Apr 13;22:277. doi: 10.1186/s13063-021-05170-3 (PMC8042906; doi:10.1186/s13063-021-05170-3)
Supplement: Supplementary file 6 — Additional file 6. Consent form. [file 13063_2021_5170_MOESM6_ESM.docx]

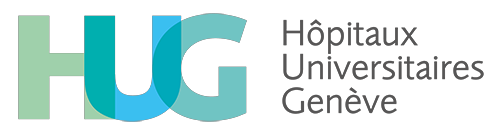


**Impact of a shared decision-making mHealth tool on caregivers’ team situational awareness, communication effectiveness, and performance during pediatric cardiopulmonary resuscitation**

**INFORMATION AND CONSENT FORM FOR A PERSON PARTICIPATING IN BIOMEDICAL RESEARCH**

As part of a study to compare the impact of a shared decision-making mHealth tool on caregivers’ team situational awareness, communication effectiveness, and performance during standardized, simulation-based, pediatric in-hospital resuscitation scenarios with conventional methods, I have been invited to take part in this study by the study investigators.

In order to give informed consent, I received and understood the following information:

- A brief description of the research, its purpose and the expected benefits.

- The chronology of the different study phases and the total duration of my participation.

- My hierarchy accepts my participation to this study during my working hours.

- I will be randomly allocated by chance to the mHealth intervention group or the conventional method group.

- The information collected about me in this study will be anonymized (i.e. linked to a special code that is stored separately on a password-protected computer file). All information that is collected about me during the course of the research will be kept strictly confidential. No one outside the research team will have any access to any identifying information. No information relating to my skills during the study will be transmitted to my hierarchy or third parties. All identifiable information will be kept securely and will be retained for a minimum period of 5 years after the study ends.

- The anonymous results of this study may be the subject of scientific publications.

- No foreseeable risk in participating in this research is expected.

- I understand that participation in this study is entirely voluntary and I am free to refuse to take part or to withdraw from the study at any time without having to give a reason. Withdrawing from the study will not affect my future medical care or my relationship with emergency medical services or medical staff looking after me.

I confirm that I will keep strictly confidential and will not disclose or communicate to third parties, by any means whatsoever, the information that will be transmitted to me or to which I will have access during this study.

By my signature, I voluntary and freely confirm that I have read and understood the above points and therefore voluntarily and freely consent to take part in the above named study.

| ______________________ | | | _______________________ | | ________________________ | |
| --- | --- | --- | --- | --- | --- | --- |
| Participant Name | | | Date | | Signature | |
| 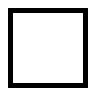 **Please initial box**   1. I have explained all the relevant aspects of the research to the participant and answered their questions. I have pointed out that participation in the research project is completely voluntary and that they may stop their participation at any time. | | | | |  |  |
| _______________________ | _______________________ | | _______________________ | |  |  |
| Individual Obtaining Consent Name | Date | | Signature | |  |  |

*When completed: 1 (original) to be kept in research record, 1 for participant, 1 for researcher site file.*
