# Supplementary material for: Impact of a shared decision-making mHealth tool on caregivers’ team situational awareness, communication effectiveness, and performance during pediatric cardiopulmonary resuscitation: study protocol of a cluster randomized controlled trial
Source: Trials. 2021 Apr 13;22:277. doi: 10.1186/s13063-021-05170-3 (PMC8042906; doi:10.1186/s13063-021-05170-3)
Supplement: Supplementary file 7 — Additional file 7. Trial information summary according to the WHO Trial Registration Data Set (Version 1.3.1). [file 13063_2021_5170_MOESM7_ESM.docx]

**Additional file 7**

| 1. Primary Registry and Trial Identifying Number | ClinicalTrials.gov: NCT04464603, <https://clinicaltrials.gov/ct2/show/NCT04464603> |
| --- | --- |
| 2. Date of Registration in Primary Registry | 9 July 2020 |
| 3. Secondary Identifying Numbers | Not applicable |
| 4. Source(s) of Monetary or Material Support | Geneva University Hospitals’ Private Foundation (QS2-25) |
| 5. Primary Sponsor | Department of Pediatric Emergency Medicine, Geneva Children’s Hospital Avenue de la Roseraie, 47, Geneva 1205, Switzerland  Contact: Professor Alain Gervaix  [Alain.Gervaix@hcuge.ch](mailto:Alain.Gervaix@hcuge.ch) |
| 6. Secondary Sponsor(s) | Not applicable |
| 7. Contact for Public Queries | **Doctor Johan N. Siebert**  Department of Pediatric Emergency Medicine, Geneva Children’s Hospital Avenue de la Roseraie, 47, Geneva 1205, Switzerland  [Johan.Siebert@hcuge.ch](mailto:Johan.Siebert@hcuge.ch) |
| 8. Contact for Scientific Queries | **Doctor Johan N. Siebert**  Department of Pediatric Emergency Medicine, Geneva Children’s Hospital Avenue de la Roseraie, 47, Geneva 1205, Switzerland  [Johan.Siebert@hcuge.ch](mailto:Johan.Siebert@hcuge.ch) |
| 9. Title | Impact of a shared decision-making mHealth tool on caregivers’ team situational awareness, communication effectiveness, and performance during pediatric cardiopulmonary resuscitation: study protocol of a randomized controlled trial |
| 10. Scientific Title | Impact of a shared decision-making mHealth tool on caregivers’ team situational awareness, communication effectiveness, and performance during pediatric cardiopulmonary resuscitation: study protocol of a randomized controlled trial |
| 11. Countries of Recruitment | Switzerland |
| 12. Health Condition(s) or Problem(s) Studied | Situational awareness of rescuers during pediatric cardiopulmonary resuscitation |
| 13. Intervention(s) | Intervention arm: simulated pediatric cardiopulmonary resuscitation supported by an integrative and shared mHealth device, namely “InterFACE”  Control arm: conventional methods not supported by an mHealth device |
| 14. Key Inclusion and Exclusion Criteria | See main body of protocol |
| 15. Study Type | An interventional, single center, randomized controlled parallel trial, with participants blinding and using a single, constant 1:1 allocation ratio determined with web-based software |
| 16. Date of First Enrollment | Anticipated April 1st 2021 |
| 17. Sample Size | 36 |
| 18. Status | Pending |
| 19. Primary Outcome | Situational awareness using SAGAT instrument during pediatric CPR |
| 20. Key Secondary Outcomes | Leadership (RTLE instrument), Team performance (TEAM instrument), Time to critical life-saving maneuvers, Medication dosage errors, Errors in number of shocks and energy doses, Usability of the mHealth device (SUS instrument), Users perceived stress, Users Satisfaction |
| 21. Review | Declaration of no objection by SwissEthics (Req-2020-00294) |
| 24. sharing statement | An anonymous copy of the final datasets underlying publications resulting from this study will be available from the corresponding author upon reasonable and approved request |
